# Crosstalk of Synapsin1 palmitoylation and phosphorylation controls the dynamicity of synaptic vesicles in neurons

**DOI:** 10.1038/s41419-022-05235-4

**Published:** 2022-09-12

**Authors:** Peipei Yan, Huicong Liu, Tao Zhou, Pu Sun, Yilin Wang, Xibin Wang, Lin Zhang, Tian Wang, Jing Dong, Jiangli Zhu, Luxian Lv, Wenqiang Li, Shiqian Qi, Yinming Liang, Eryan Kong

**Affiliations:** 1 The Second Affiliated Hospital of Xinxiang Medical University, Xinxiang, China; 2 Institute of Psychiatry and Neuroscience, Xinxiang key laboratory of protein palmitoylation and major human diseases, Xinxiang Medical University, Xinxiang, China; 3 Department of Urology, State Key Laboratory of Biotherapy and Cancer Center, West China Hospital, Sichuan University and National Collaborative Innovation Center, 610041 Chengdu, China; 4 Laboratory of Genetic Regulators in the Immune System, Henan Key Laboratory of Immunology and Targeted Therapy, School of Laboratory Medicine, Xinxiang Medical University, Xinxiang, China

**Keywords:** Cellular neuroscience, Sumoylation

## Abstract

The dynamics of synaptic vesicles (SVs) within presynaptic domains are tightly controlled by synapsin1 phosphorylation; however, the mechanism underlying the anchoring of synapsin1 with F-actin or SVs is not yet fully understood. Here, we found that Syn1 is modified with protein palmitoylation, and examining the roles of Syn1 palmitoylation in neurons led us to uncover that Syn1 palmitoylation is negatively regulated by its phosphorylation; together, they manipulate the clustering and redistribution of SVs. Using the combined approaches of electron microscopy and genetics, we revealed that Syn1 palmitoylation is vital for its binding with F-actin but not SVs. Inhibition of Syn1 palmitoylation causes defects in SVs clustering and a reduced number of total SVs in vivo. We propose a model in which SVs redistribution is triggered by upregulated Syn1 phosphorylation and downregulated Syn1 palmitoylation, and they reversibly promote SVs clustering. The crosstalk of Syn1 palmitoylation and phosphorylation thereby bidirectionally manipulates SVs dynamics in neurons.

## Introduction

The intrinsic nature of Synapsin1 (Syn1) ensures high binding affinity with various interacting partners, in particular, synaptic vesicles (SVs) [1, 2] and F-actin [3, 4] within presynaptic domains, whereby they play essential roles in regulating the dynamic recycling of the SVs, including the clustering and release of the localized vesicle pools, and thus control the transmission of action potentials [5]. Underlying this phenomenon, the mechanism is the incoming action potentiation inducing Ca^2+^-dependent phosphorylation of Syn1, which results in the disassociation of Syn1 with its interacting proteins and the release of SVs [5, 6]. Considering that the assembly and release of the SVs is precisely controlled in response to consistently changing stimuli at the presynaptic locus, the regulatory mechanism might require more sophisticated molecular actions beyond the phosphorylation of Syn1 alone. However, such a refined mechanism has yet to be discovered.

To identify potential palmitoylated proteins in the central nervous system (CNS) by palm-proteomics in our lab [7, 8], Syn1 was observed, suggesting that Syn1 is possibly modified with protein palmitoylation [8, 9]. In principle, the reversible protein S-palmitoylation reinforces the hydrophobicity of the subdomain of a given protein that is palmitoylated, and thereby facilitates its binding with other motifs, such as subcellular membranes and cytoskeletons. [10, 11]. Remarkably, the dynamic nature of this modification can be recycled between protein palmitoylation (catalyzed by DHHC1-24) and depalmitoylation (catalyzed by APT1/2, PPT1/2, and ABHD17a), which solely occurs on cysteine residues [8, 12, 13].

Inspired by the findings that both protein palmitoylation and phosphorylation might occur on Syn1, we speculated that Syn1 palmitoylation might crosstalk with its phosphorylation and together participate in the regulation of the dynamics of the presynaptic vesicle pool in vivo. Indeed, we showed that Syn1 is palmitoylated by DHHC5 in neurons. Notably, phosphorylated Syn1 negatively regulates its palmitoylated form, and they are both required for the proper assembly and redistribution of SVs on and off cytoskeletons as F-actin within presynaptic domains.

## Results

### Syn1 is palmitoylated at cys-223, cys-360, and cys-370

**Fig. 1:**
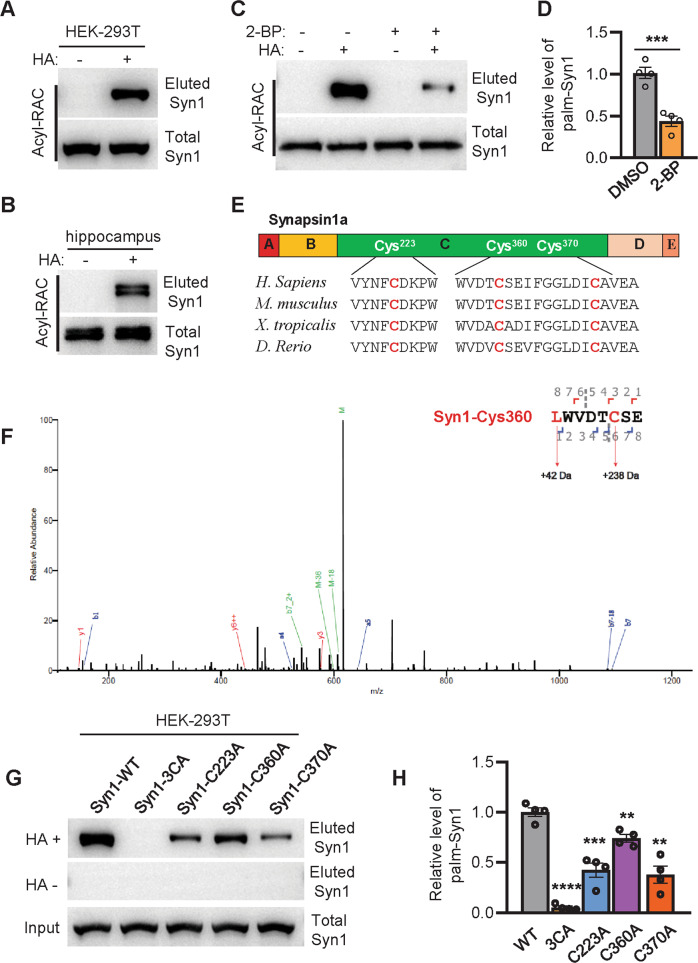
Syn1 is palmitoylated at Cys-223, Cys-360, and Cys-370. **A**, **B** Syn1 expressed in HEK-293T cells or lysate of mice hippocampi was analyzed for protein palmitoylation by Acyl-RAC assay. HA+, with NH_2_OH, HA-, without NH_2_OH. **C**, **D** HEK-293T cells expressing Syn1-his was incubated with 50 μM 2-BP for 8 h and evaluated for the level of palm-Syn1. ***p < 0.001; *t*-test (*n* = 4 biological replicates). **E** Protein sequences of Syn1 from various species were aligned for analyzing cysteine conservation. **F** Purified Syn1-flag was probed by mass-spectrometry, a mass-shift of 238 Da linked to cysteine is a hallmark for palmitoylation, suggesting that cysteine-360 is palmitoylated. **G**, **H** His-tagged Syn1-WT and its mutants were analyzed by Acyl-RAC for the level of palm-Syn1. **p < 0.01; ***p < 0.001; ****p < 0.0001; one-way ANOVA; *n* = 4 biological replicates, Data are mean ± s.e.m.

To verify whether Syn1 is palmitoylated, ectopically expressed Syn1-His in HEK-293T cells or hippocampal lysate of WT mice was subjected to the examination of protein palmitoylation by Acyl-Rac assay [8] or metabolic labeling [14]. The results demonstrated that Syn1 is modified with palmitoylation both in vitro and in vivo (Fig. 1A, B, Fig. S1A, and Supplementary File 1). For verification, 2-BP (an inhibitor of palmitoylation) was used to incubate HEK-293T cells expressing Syn1-His. Again, the results showed that 2-BP could effectively downregulate the level of Syn1 palmitoylation (palm-Syn1) (Fig. 1C, D and Supplementary File 1). Together, these experiments verified that Syn1 is palmitoylated.

As S-palmitoylation only occurs on cysteine residue, we aligned the protein sequence of Syn1 from various species and found that there are three cysteine residues available in Syn1 and that all cysteines are conserved across the different species (Fig. 1E). To determine which cysteine residue might be specifically modified with protein palmitoylation, Syn1-Flag was expressed, purified (Fig. S1B), and assessed with Mass-spectrometry (MS). The MS data showed that all three cysteines are palmitoylated, indicated by 238 Da of mass shift associated with each detected cysteine residue [8] (Fig. 1F and Fig. S1C, D). Lastly, to validate the findings from MS, various Syn1 mutants were constructed, expressed in HEK-293T cells, and evaluated for the level of palm-Syn1 by Acyl-RAC. The experiments showed that individual cysteine-mutants (cys-223, cys-360, and cys-370) could partially decrease the level of palm-Syn1, while the triple-cysteines mutant (hereafter named Syn-3CA) completely blocks Syn1 palmitoylation (Fig. 1G, H and Supplementary File 1). Thus, we concluded that Syn1 is palmitoylated at cys-223, cys-360, and cys-370.

### Syn1 palmitoylation is vital for SVs clustering in neurons

**Fig. 2:**
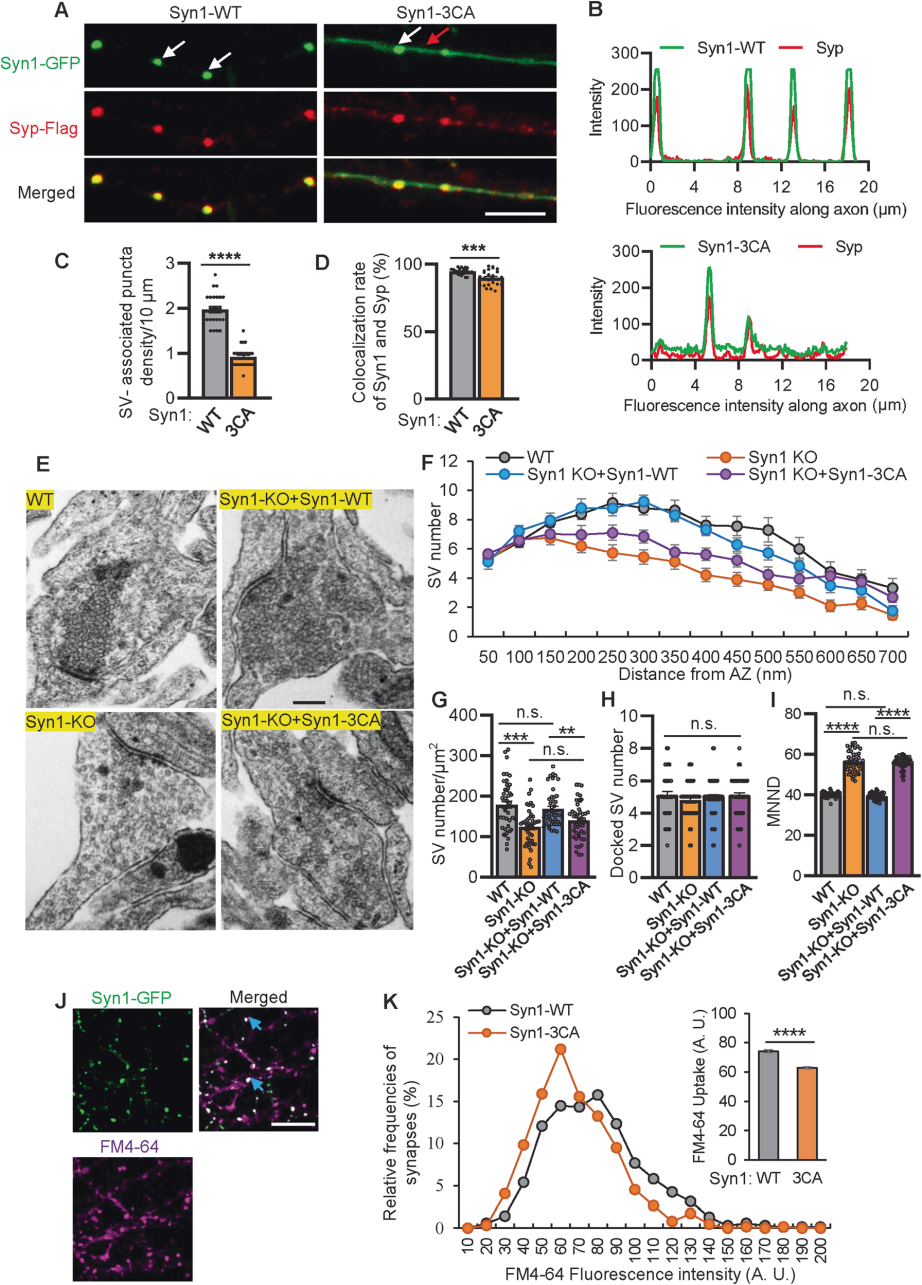
Blocking Syn1 palmitoylation affects SVs clustering. **A** Hippocampal neurons (Syn1-KO) were transfected at DIV8-9 to express Syp-Flag and Syn1-GFP or Syn1-3CA-GFP, and fixed at DIV15-16 for imaging. Synaptophysin (Syp) is a marker of SV cluster. Scale bar, 5 μm. **B**–**D**, the fluorescence intensity along axon (**B**), the density of SV-associated puncta (**C**, *n* = 25 ROI ., ***p < 0.001) and the colocalization (Pearson coefficient) of Syp and Syn1 were quantified (**D**, *n* = 25 ROI from 4 biological repeats, ***p < 0.001). **E** Representative electron micrographs of excitatory terminals of WT, Syn1-KO and Syn1-KO hippocampal neuron expressing Syn1 or Syn1-3CA. Scale bar, 200 nm. **F** Quantification of the number of synaptic vesicles located within 700-nm-wide compartments centered at the active zone of WT, Syn1-KO and Syn1-KO neuron expressing GFP-Syn1-WT or Syn1-3CA (*n* = 40-41 synapses from 3 mice). **G**–**I**, The SV density (WT vs Syn1-KO, ***p < 0.001; Syn1-KO + Syn1-WT vs Syn1-KO + Syn1-3CA, **p < 0.01), number of docked vesicles (*p* > 0.1) and SV clustering (mean nearest neighbor distance (MNND), WT vs. Syn1-KO, ****p < 0.0001; Syn1-KO + Syn1-WT vs. Syn1-KO + Syn1-3CA, ****p < 0.0001) were quantified (*n* = 44–46 synapses from 3 mice, one-way ANOVA). **J** Neurons expressing GFP-Syn1 were exposed to 55 mM KCl for uptake of FM4-64. Scale bar, 10 μm. **K** Measuring FM4-64 uptake in Syn1-GFP-positive synapses. (n = 642-705 ROI from 4 biological repeats, ****p < 0.0001). n.s., not significant. Data are mean ± s.e.m.

To examine if palm-Syn1 is involved in regulating SVs clustering in neurons, we expressed GFP-tagged Syn1-WT and Syn1-3CA proteins in hippocampal neurons isolated from Syn1-KO mice (Fig. S2A–D). The experiments showed that the expression of Syn1 displays a puncta-shaped but disconnected distribution of SVs (indicative of clustered SVs) along the axonal shaft, while the distribution of Syn1-3CA is partially scattered and diffused (Fig. 2A, B). Accordingly, the quantification data indicated that the number of SV-associated puncta, as well as the colocalization rate of Syn1 and Synaptophysin (Syp, a marker of SVs), along the axonal shaft are significantly decreased in Syn1-3CA expressing neurons compared to the cells expressing Syn1-WT (Fig. 2C, D), indicating that Syn1 palmitoylation is required for proper SVs clustering in presynaptic domains.

To understand the biological nature of the impaired SVs clustering induced by the expression of non-palmitoylated Syn1 (Syn1-3CA), WT and Syn1-KO neurons were cultured and the latter was infected with lentiviruses expressing GFP-tagged Syn1-WT or Syn1-3CA, and then subjected to electron microscopy analysis (Fig. 2E). The results showed that in the proximity (200–400 nm) of the active zone (AZ) the number of SVs was decreased in the terminals of Syn1-KO neurons or rescuing Syn1-KO neurons with Syn1-3CA as compared to the WT or rescuing Syn1-KO neurons with Syn1-WT (Fig. 2F, G). Most intriguingly, the mean nearest neighbor distance (MNND, characterizing the density of SVs/distance among SVs) of SVs was markedly increased in Syn1-KO or Syn1-3CA expressing neurons as compared to the WT and Syn1-WT expressing neurons (Fig. 2I). However, the number of docked SVs (readily released SVs) did not vary detectably among these groups (Fig. 2H). For further verification in vivo, we attempted to generate Syn1-3CA-KI mice; unfortunately, the mutant protein Syn1-3CA expresses at a minimum level in Syn1-KI mice for unknown reasons and thus precluded further analysis.

Last, to briefly test if the impaired SVs clustering in neurons expressing Syn1-3CA might affect cellular functions, the FM4-64 was loaded into infected neurons using high K + depolarization to induce multiple rounds of exo-endocytosis, and thus the entire pool of recycling SVs was labeled, identified by the presence of Syn1-GFP (Fig. 2J). The results indicated that the expression of Syn1-3CA greatly reduces the capacity of SV recycling as compared to cells expressing Syn1-WT (Fig. 2K). Taken together, these results showed that Syn1 palmitoylation is vital for SVs clustering in neurons, and reducing the level of palm-Syn1 (Syn1-3CA) causes loosely-compacted SVs and a decreased number of total SVs.

### ZDHHC5-mediated Syn1 palmitoylation is involved in regulating SVs clustering in vivo

**Fig. 3:**
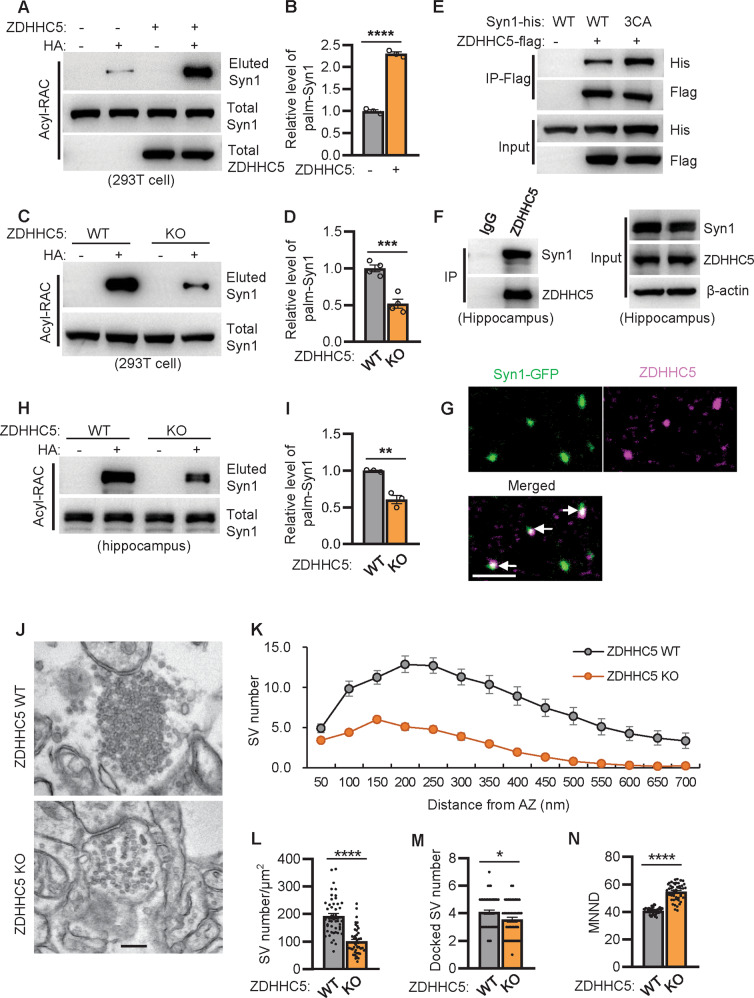
ZDHHC5-mediated Syn1 palmitoylation is likely involved in regulating SVs clustering. **A**, **B** ZDHHC5 was coexpressed with Syn1 in HEK-293T cells and evaluated for the level of palm-Syn1 (*n* = 3 biological replicates, ****p < 0.0001). HA + , with NH_2_OH, HA-, without NH_2_OH. **C**, **D** Syn1 was expressed in WT and ZDHHC5-KO cells and examined for the level of palm-Syn1 (*n* = 4 biological replicates, ***p < 0.001). **E** Syn1-WT or Syn1-3CA was coexpressed with or without ZDHHC5 in HEK-293T cells for coimmunoprecipitation assays. **F** Lysate of mouse hippocampus was used for precipitate Syn1 by using ZDHHC5 antibody. **G** Hippocampal neurons were transfected with GFP-Syn1 and fixed for imaging. Scale bar, 5 μm. **H**–**I** Hippocampus lysates of WT and ZDHHC5-KO mice were processed with Acyl-RAC for palm-Syn1 (*n* = 3 biological replicates, **p < 0.01). **J** Representative electron micrographs of excitatory terminals of hippocampal neurons from WT and ZDHHC5-KO mice. Scale bar, 200 nm. **K** Quantification of the number of synaptic vesicles located within 700-nm-wide compartments centered at the active zone of synapses from WT and ZDHHC5-KO mice (*n* = 50–51 synapses from 3 mice). **L**–**N** SV density (*n* = 46 synapses from 3 mice, ****p < 0.0001), Number of docked vesicles (*n* = 50 synapses from 3 mice, *p = 0.0207), and MNND (mean nearest neighbor distance); *n* = 49–53 synapses from 3 mice, ****p < 0.0001) were quantified in WT and ZDHHC5-KO mice. Data are mean ± s.e.m.

As S-palmitoylation is dynamic, we co-expressed all known ZDHHCs with Syn1 to identify the enzymes that might catalyze Syn1 palmitoylation. The initial screening identified ZDHHC5, ZDHHC15, and ZDHHC19 as possible candidates for Syn1 palmitoylation (Fig. 3A, B, Fig. S3A, B, and Supplementary File 1). RT-PCR indicated that zdhhc5 mRNA is abundantly expressed in the adult mouse hippocampus (Fig. S3C). For verification, ZDHHC5 was deleted in HEK-293T cells (ZDHHC5-KO, Fig. S4A–C), and the RAC assay showed that the level of palm-Syn1 is dramatically downregulated in ZDHHC5-KO as compared to the WT cells (Fig. 3C, D and Supplementary File 1). Additionally, ZDHHC5 coprecipitates with Syn1 or Syn1-3CA when both proteins are expressed in HEK-293T cells; surprisingly, Syn1-3CA enhances its binding with ZDHHC5 over that of the WT Syn1 (Fig. 3E and Supplementary File 1). Most importantly, endogenously expressed Syn1 and ZDHHC5 coprecipitate (Fig. 3F and Supplementary File 1) and ectopically expressed Syn1 colocalizes with endogenous ZDHHC5 in cultured hippocampal neurons (Fig. 3G).

To confirm that ZDHHC5 catalyzes Syn1 palmitoylation in neurons, ZDHHC5 was depleted in C57BL6 mice (ZDHHC5-KO, Fig. S4D, G). The RAC assay showed that Syn1 is readily palmitoylated in WT mice, while the level of palm-Syn1 is apparently suppressed in ZDHHC5-KO mice (Fig. 3H, I and Supplementary File 1). Considering that a decreased level of palm-Syn1 induces severe defects in SVs clustering ex vivo (Fig. 2E–I), we elected to evaluate the status of SV clustering in the hippocampus of ZDHHC5-KO mice. Images from electron microscopy showed that the depletion of ZDHHC5 remodels SVs clustering in various ways (Fig. 3J) *i.e*., the number of total SVs and docked SVs (Fig. 3K–M), and that the density of SVs associated with AZ (Fig. 3L) is significantly downregulated in ZDHHC5-KO mice as compared to the WT mice, while the MNND of SVs was considerably increased in zDHHC5-KO as compared to WT mice (Fig. 3N). Together, these findings demonstrated that ZDHHC5 catalyzes Syn1 palmitoylation, and the absence of ZDHHC5 results in severe defects in SVs clustering in hippocampal neurons in vivo, indicated by less compacted SVs and decreased numbers of total SVs at the presynapse.

### Syn1 palmitoylation facilitates its binding with F-actin but not SVs

**Fig. 4:**
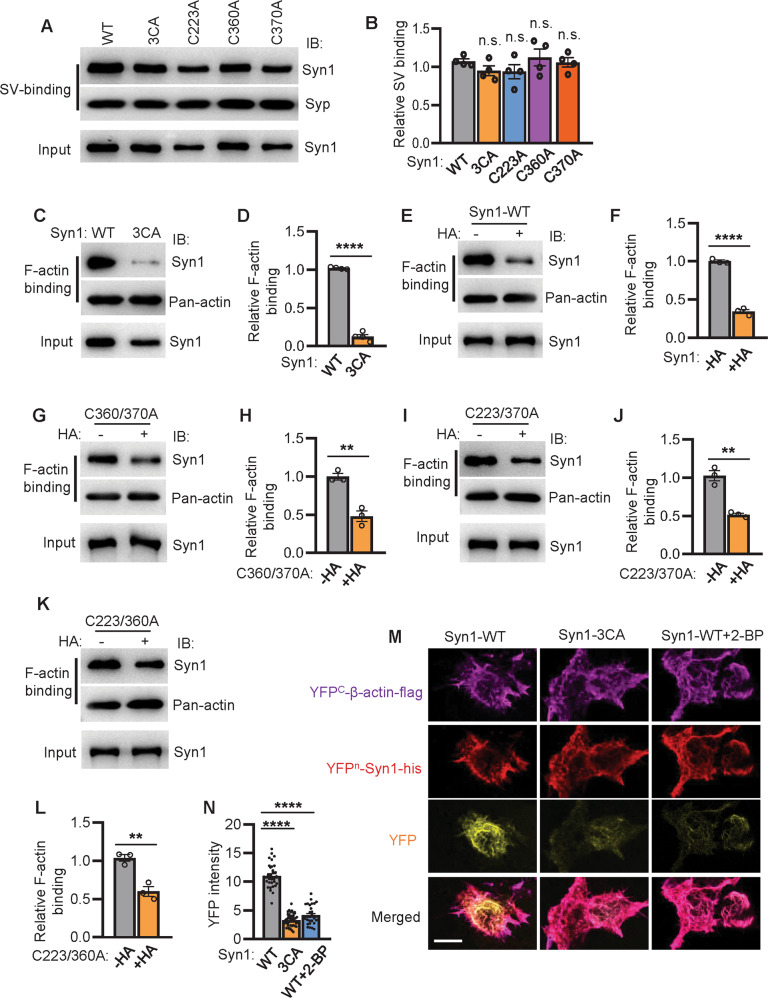
Syn1 palmitoylation facilitates its binding with F-actin but not SVs. **A**, **B** SVs were extracted from WT mice brain and incubated with purified Syn1-flag or its mutants for affinity-binding assay. *p* > 0.1, one-way ANOVA, *n* = 4 biological replicates. **C**, **D** purified Syn1-WT and Syn1-3CA were incubated with actin and subjected for F-actin-binding assay (*n* = 4 biological replicates, ****p < 0.0001). **E**, **F** purified Syn1-flag treated with or without HA (0.5 M for 2 h) was subjected for F-actin-binding assay (*n* = 3 biological replicates, ****p < 0.0001). **G**–**L** purified Syn1-C360/370 A (**G**, **H**, **p = 0.0030), Syn1-C223/370 A (**I**, **J**, **p = 0.0018), or Syn1-C223/360 A (**K**, **L** **p = 0.0066) were treated with or without HA (0.5 M for 2 h) and subjected for F-actin-binding assays (*n* = 3 biological replicates). **M**, **N** YFP^n^-Syn1-WT and YFP^n^-Syn1-3CA were coexpressed with YFP^c^-β-actin in HEK-293T cell for BiFC assays and the YFP fluorescence was quantified (*n* = 31–46 cells from 4 biological repeats). Scale bar, 10 μm. One-way ANOVA, ****p < 0.0001. n.s., not significant. Data are mean ± s.e.m.

To understand how SVs clustering is regulated by Syn1 palmitoylation, we tested the possibilities whereby palmitoylation might modulate the interactions of Syn1 with SVs or F-actin [4]. First, we examined if palm-Syn1 is involved in regulating its binding with SVs. Purified synapsin-depleted SVs (Fig. S6A) were incubated with purified Syn1 or its mutants (Syn1-3CA, C223A, C360A and C370A) by FLAG-affinity purification. The SVs were pelleted by ultracentrifugation and subjected to western blotting to determine the amount of bound-Syn1 in the SV fraction. The results showed that manipulating the level of palm-Syn1 does not significantly alter the binding affinity of Syn1 with SVs (Fig. 4A, B and Supplementary File 1). Similarly, it was also shown that altering the level of palm-Syn1 by either expressing or deleting ZDHHC5 in HEK-293T cells does not apparently affect the association of Syn1 with lipid membranes (Fig. S5A). Second, we examined if palmitoylation is required for the interaction of Syn1 with F-actin. Strikingly, blocking Syn1 palmitoylation (Syn1-3CA, purified) markedly inhibited its binding with F-actin, as compared to the Syn1-WT (Fig. 4C, D and Supplementary File 1). To further validate this finding, hydroxylamine (HA) was applied to remove palmitoylation from Syn1. Accordingly, purified Syn1 (with palmitoylation, Fig. S6B), treated with or without HA, was incubated with F-actin for affinity measurements [4]. Consistently, the results confirmed that a decreased level of palm-Syn1 markedly attenuated its interaction with F-actin (Fig. 4E, F and Supplementary File 1).

To determine the contribution of palmitoylated-cysteine in Syn1 from the perspective of its binding affinity with F-actin, a set of Syn1 mutants were expressed, purified, and incubated with F-actin for affinity measurements. The results showed that palmitoylated-cysteine in Syn1 is required for sustaining the full affinity to associate with F-actin, as different combinations of cysteine-mutations significantly decrease its interaction with F-actin (Fig. 4G–L and Supplementary File 1). Additionally, bimolecular fluorescence complementation assay [15] (BiFC or Split YFP, with YFP^n^-Syn1 (1) + YFP^c^-β-actin (3) for BiFC, Fig. S5B, C) was carried out to visualize the binding of Syn1 with F-actin ex vivo. The fluorescence images demonstrated that while Syn1-WT interacts with β-actin and illuminates YFP, decreasing the level of palm-Syn1 by either cysteine mutations (Syn1-3CA or other mutants) or treatment with 2-BP greatly alleviated YFP illumination *i.e*., the interaction of Syn1 with β-actin was impaired (YFP-channel, Fig. 4M, N and Fig. 6C, D). Together, these experiments indicated that palmitoylation facilitates the direct interaction of Syn1 with F-actin but not SVs, through which it might manipulate the dynamics of SVs.

### Syn1 palmitoylation is negatively regulated by its site 1 phosphorylation

**Fig. 5:**
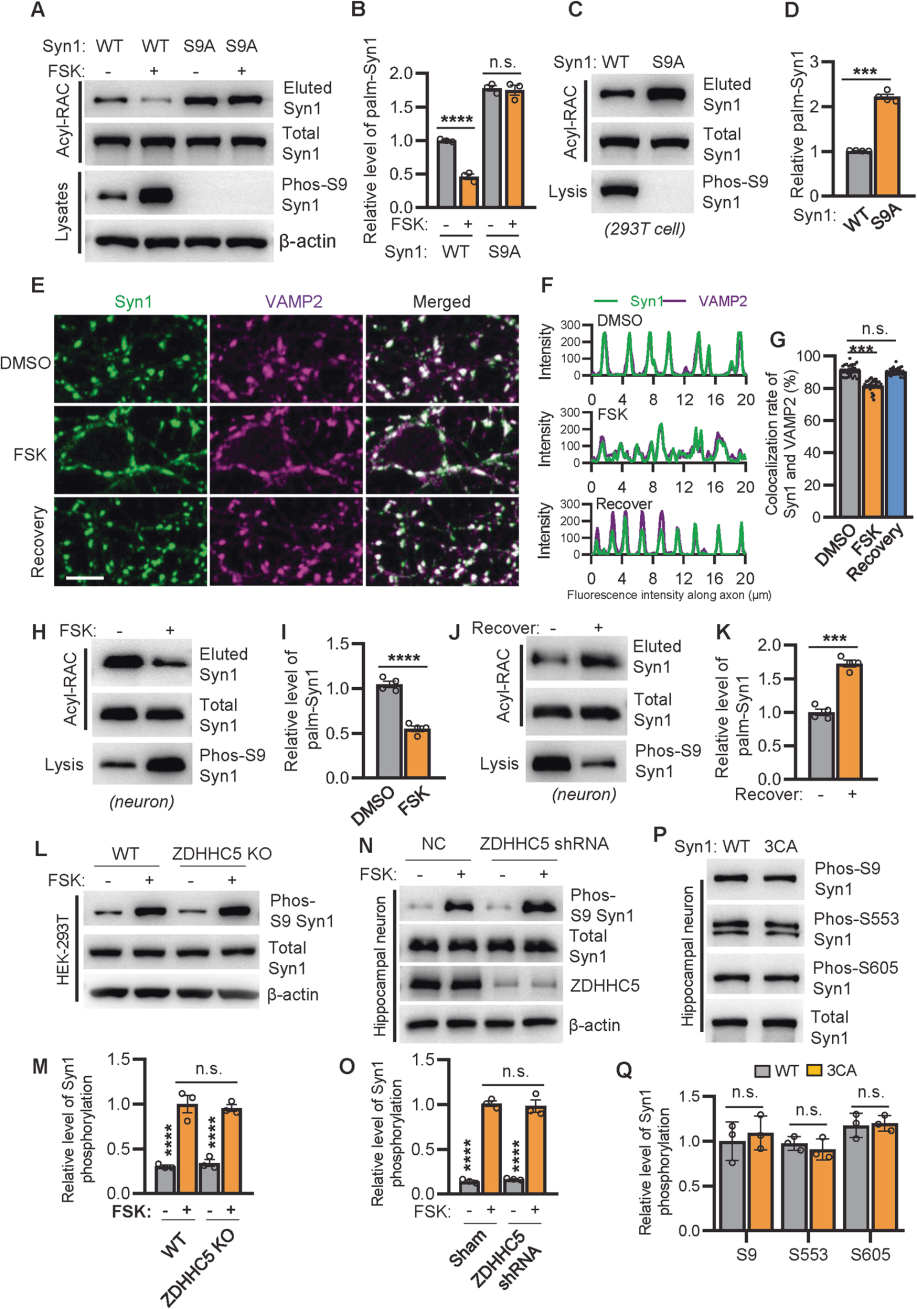
Syn1 palmitoylation is negatively regulated by its phosphorylation. **A**, **B** HEK-293T cells expressing Syn1 or Syn1-S9A were treated with or without FSK (10 μM) for 30 min and analyzed for the level of palm-Syn1. (*n* = 4 biological replicates, ****p < 0.0001). **C**, **D** HEK-293T cells expressing Syn1-WT or Syn1-S9A were evaluated for the level of palm-Syn1 (*n* = 4 biological replicates, ***p = 0.0002). **E** cultured hippocampal neurons (DIV15) were treated with DMSO or FSK for 30 min, for the recovery group, FSK was washed out and recover for 1 h, before fixation for imaging. VAMP2 is a marker of SV cluster. Scale bar, 5 μm. **F**, **G** the fluorescence intensity of Syn1 and VAMP2 along axon shaft was profiled (**F**) and the colocalization rate of Syn1 and VAMP2 was quantified (*n* = 32 synapses from 4 biological repeats, one-way ANOVA, DMSO vs FSK, ***p < 0.001) (**G**). **H**, **I** hippocampal neurons treated with or without FSK were analyzed for the level of palm-Syn1 (*n* = 4 biological replicates, ****p < 0.0001). **J**, **K** FSK-treated neurons with or without recovery (FSK washout for 1 h) were evaluated for the level of palm-Syn1 (*n* = 4 biological replicates, ***p = 0.0002). **L**, **M** WT or ZDHHC5-KO HEK-293T cells were treated with or without FSK for analyzing the level of phos-S9 Syn1 (n = 3 biological replicates, FSK- vs. FSK + , ****p < 0.0001; WT + FSK vs. ZDHHC5-KO + FSK, *p* = 0.99). **N**, **O** WT hippocampal neurons were transfected with sham or ZDHHC5-shRNA and subjected for the analysis of phos-S9 Syn1 (*n* = 3 biological replicates, FSK- vs. FSK + , ****p < 0.0001; NC + FSK vs ZDHHC5-shRNA+FSK, *p* = 0.99). **P**, **Q** hippocampal neurons infected with lentiviruses to express Syn1-WT or Syn1-3CA were analyzed for Syn1 phosphorylation (*n* = 3 biological replicates). n.s., not significant. Data are mean ± s.e.m.

We showed that the reduction of Syn1 palmitoylation leads to the diffusion of SVs in neurons (Fig. 2A–I and Fig. 3J–N), a phenomenon that is comparable to the effect of Syn1 phosphorylation at site 1 (serine-9, hereafter referred as phospho-Syn1) on SVs mediated by PKA, which can be activated by Forskolin (FSK) [16–18] (Fig. S7A, B). We therefore tested if FSK-induced upregulation of phospho-Syn1 would modulate the level of palm-Syn1. Interestingly, results from HEK-293T cells showed that as FSK augments the level of phospho-Syn1, the level of palm-Syn1 is readily downregulated (Fig. 5A, B and Supplementary File 1). Conversely, as serine-9 was mutated into alanine (S9A) to block site 1 phosphorylation in Syn1, the level of palm-Syn1 was significantly increased in cells expressing Syn1-S9A, compared to that of cells expressing Syn1 (Fig. 5C, D and Supplementary File 1). However, mutating Serine553, Ser568, and Ser605 phosphorylation in Syn1 does not impair Syn1 palmitoylation (Fig. S7H, I). Notably, FSK fails to modulate the level of palm-Syn1 if site 1 phosphorylation is blocked in Syn1 (Fig. 5A, panels 3, 4), indicating that FSK-induced downregulation of palm-Syn1 cannot bypass Syn1-S9 phosphorylation. Results from cultured hippocampal neurons verified that FSK increases the level of phospho-Syn1, decreases the level of palm-Syn1 (Fig. 5H, I and Supplementary File 1), and causes the redistribution of SVs, characterized by broader distribution of Syn1 fluorescence signal and decreased colocalization of Syn1 and VAMP2 (Fig. 5E–G). Most critically, this process is highly dynamic, as the level of phospho-Syn1 decreased when FSK was washed out and the cultured hippocampal neurons were left to recover for 1 h (Fig. S7C, D), and the level of palm-Syn1 was again upregulated (Fig. 5J, K and Supplementary File 1) and SVs re-clustered (Fig. 5E–G, panel: Recovery).

Next, we hypothesized that alteration of the level of palm-Syn1 would affect its phosphorylation. Accordingly, either the expression of ZDHHC5 was reduced (Fig. S7E, G) or Syn1-3CA was expressed to evaluate the level of phospho-Syn1. The results showed that Syn1 phosphorylation (Serine9, Serine553, and Ser605) were not detectably altered irrespective of palmitoylation status (Fig. 5L–Q and Supplementary File 1). In summary, these results show that Syn1 phosphorylation negatively regulates its palmitoylated form but not the reverse; the redistribution of SVs is possibly regulated by the sequential activities of upregulated phospho-Syn1 and downregulated palm-Syn1 in neurons.

### FSK-triggered SVs release depends on the downregulation of Syn1 palmitoylation

**Fig. 6:**
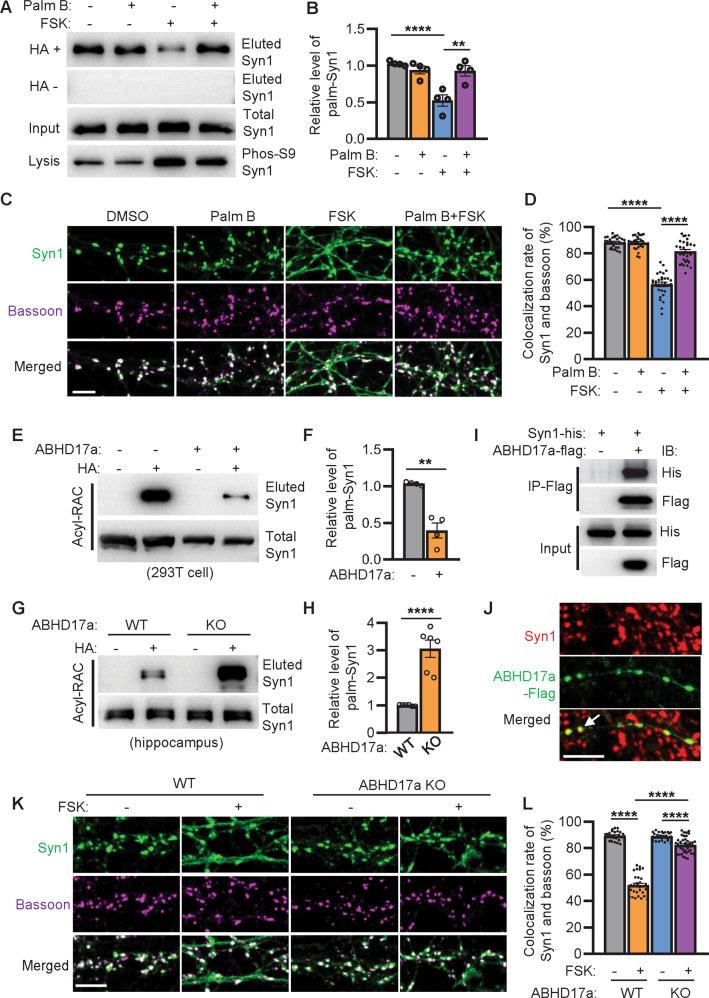
FSK triggered SVs release depends on the downregulation of Syn1 palmitoylation. **A**, **B** hippocampal neurons (DIV15) treated with either FSK (10 μM) or Palm B (10 μM) were analyzed for protein palmitoylation by Acyl-RAC assay. (*n* = 4 biological replicates; One-way ANOVA, DMSO *vs*. FSK, ****p < 0.0001; FSK vs. Palm B + FSK, **p = 0.002). **C**, **D** hippocampal neurons treated with FSK or Palm B was fixed for imaging. Scale bar, 5 μm. The colocalization rate of Syn1 and bassoon was quantified (*n* = 30 ROI from 4 biological replicates; One-way ANOVA, DMSO vs. FSK, ****p < 0.0001; FSK vs. Palm B + FSK, ****p < 0.0001). **E**, **F** ABHD17a was expressed with Syn1 in HEK-293T cells and evaluated for the level of palm-Syn1 (*n* = 4 biological replicates, **p = 0.0078). **G**, **H** lysates of WT and ABHD17a-KO mice hippocampi were evaluated for the level of palm-Syn1 (*n* = 4 biological replicates, ****p < 0.0001). **I** Syn1-his and ABHD17a-flag were expressed in HEK-293T cells and tested for immune-coprecipitation. **J** hippocampal neurons expressing flag-ABHD17a were fixed for colocalization analysis with endogenous Syn1. Scale bar, 5 μm. **K**, **L** hippocampal neurons isolated from WT and ABHD17a-KO mice were treated with or without FSK and stained with Syn1 and Bassoon for colocalization analysis. (*n* = 27–43 ROI from 4 biological repeats, WT vs. WT-FSK, ****p < 0.0001; WT-FSK vs. KO-FSK, ****p < 0.0001). Scale bar, 5 μm. Data are mean ± s.e.m.

As FSK induces the upregulation of phospho-Syn1 and the downregulation of palm-Syn1(Fig. 5A–K), the latter might induce the disassociation of Syn1 from F-actin (Fig. 4C–N), and thus the redistribution of SVs (Fig. 5E–G). We therefore tested if FSK-induced SVs release could be inhibited by PalmB treatment, as PalmB inhibits depalmitoylation [8, 9] and would possibly hinder the downregulation of palm-Syn1. Accordingly, we treated cultured hippocampal neurons with PalmB and FSK, alone and in combination. The results showed that while FSK lowers the level of palm-Syn1 upon the activation of phospho-Syn1 (Fig. 6A, lane 3, and Supplementary File 1), the combined treatment with both PalmB and FSK sustained relatively higher levels of both palm-Syn1 and phospho-Syn1 (Fig. 6A, B, lane 4), indicating that FSK-induced downregulation of palm-Syn1 is not an autonomous event, but rather depends on certain unknown thioesterases (catalyze depalmitoylation) in neurons. At the same time, cultured hippocampal neurons were also treated with FSK and PalmB. The immunofluorescence images clearly indicate that FSK-triggered SVs redistribution (Fig. 6C, D, panel 3) is indeed inhibited by the combined treatment with FSK and PalmB (Fig. 6C, D, panel 4) as compared to the treatments with PalmB or DMSO alone (Fig. 6C, D, panel 1 and 2). In brief, these experiments support the hypothesis that FSK-induced SVs release depends on the downregulation of palm-Syn1.

To identify the enzyme that might catalyze the depalmitoylation of Syn1, Syn1 was co-expressed in HEK-293T cells with all known thioesterases for screening. The RAC assays showed that PPT1/2 and ABHD17a could effectively reduce the level of palm-Syn1 (Fig. 6E, F, Fig. S8A, B, and Supplementary File 1). Further investigations suggested that ABHD17a (mRNA) is dominantly expressed in hippocampal neurons (Fig. S8C) and deficiency of PPT1 in vivo (PPT1-KI mice [8, 19]) does not significantly change the level of palm-Syn1 in the mouse hippocampus (Fig. S8H). To further validate this, ABHD17a was depleted in either HEK-293T cells (Fig. S9A–C) or in mice (ABHD17a-KO, Fig. S10A–C). The results showed that the level of palm-Syn1 was augmented in both cases as compared to that of the WT control (Fig. 6G, H, Fig. S8D, E, and Supplementary File 1). Moreover, we illustrated that immunoprecipitated Syn1 could pull down ABHD17a when both proteins are co-expressed (Fig. 6I and Supplementary File 1), and critically, endogenously expressed Syn1 colocalizes with ABHD17a in hippocampal neurons (Fig. 6J). Together, this evidence supports ABHD17a catalyzing Syn1 depalmitoylation in vivo.

Next, we examined the possibility that the removal of ABHD17a in neurons might hinder the process of SVs redistribution induced by FSK as well. While FSK upregulates the level of phospho-Syn1, it fails to downregulate the level of palm-Syn1 in ABHD17a-KO neurons (Fig. S8F, G). Similarly, as FSK facilitates SVs redistribution in WT neurons, this process is inhibited in ABHD17a-KO neurons (Fig. 6K, L). To further illustrate the altered dynamics of Syn1 palmitoylation in ABHD17a-KO neurons by metabolic labeling, cultured hippocampal neurons were incubated with 17-ODYA and treated with FSK for different periods. The results showed that upon FSK treatment, palm-Syn1 was downregulated in WT neurons; however, such dynamicity was abolished in ABHD17a-KO neurons (Fig. S9F). Together, this showed that FSK-induced SVs release depends on the depalmitoylation of Syn1, which is potentially mediated by ABHD17a at the presynaptic locus.

## Discussion

**Fig. 7:**
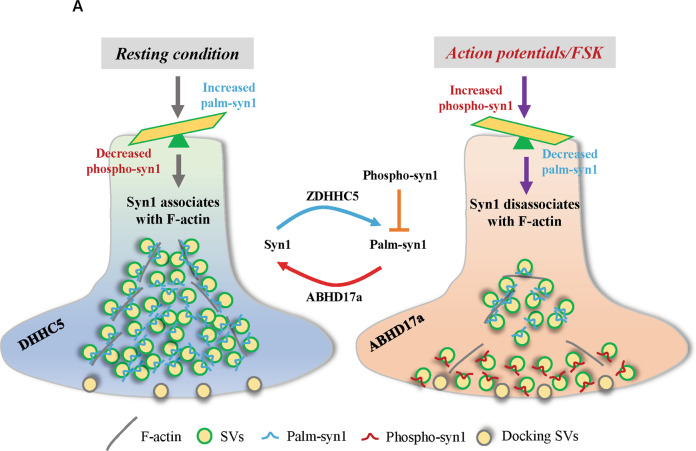
Schematic presentation of SVs dynamicity regulated by the crosstalk of Syn1 palmitoylation and phosphorylation within presynapse. **A** under resting condition, downregulated Syn1 phosphorylation upregulates its level of palmitoylation mediated by ZDHHC5, together they ensure the high binding affinity of Syn1 with SVs and F-actin to maintain compacted SVs pool. In action potentiation (or triggered by FSK), Syn1 phosphorylation is upregulated, which then downregulates the level of palm-Syn1 potentially catalyzed by ABHD17a, together they trigger the disassociation of Syn1 with F-actin (partially) and thus the diffusion of SVs within presynapse. Of note, Syn1 phosphorylation negatively regulates its level of palmitoylation but not reversely. Moreover, the number of docking SVs are not affected within these processes.

Syn1 phosphorylation plays a central role in controlling SVs release in presynaptic domains. In the resting condition, non-phosphorylated Syn1 binds SVs and F-actin to sustain the SVs pool, while in action potentiation (or mimicked by FSK treatment) Syn1 is phosphorylated and disassociates from SVs and F-actin to mobilize SVs for exocytosis [4, 20–22]. However, how the dynamic phosphorylation of Syn1 might modulate its binding with SVs and F-actin relies on the observation that S9-phosphorylation in Syn1 might regulate its protein conformational remodeling [5, 23]. Here, we revealed that Syn1 is reversibly palmitoylated by DHHC5 and ABHD17a in vivo (Fig. 1A–H, Fig. 3A–I, and Fig. 6E–H), which act sequentially after Syn1 phosphorylation (Fig. 5A–K). Interestingly, the crosstalk of phosphorylation and palmitoylation in Syn1 controls the clustering and redistribution of SVs (Fig. 6A–L) through manipulating its binding affinity with F-actin but not SVs (Fig. 4A–M) in neurons (Fig. 7A).

There are currently two models to account for the mechanisms of SVs clustering: the classical scaffold model, in which vesicles are proposed to be anchored via actin filaments [22, 23], and the more recent liquid phase model, in which liquid–liquid phase separation (LLPS) mediates clustering almost exclusively via the D region (IDR) of synapsin [24, 25]. A weakness of the scaffold model is that actin filaments are scarce or absent within the vesicle cluster, but rather accumulate around the cluster. Nevertheless, the current data lend support to a role of synapsin1-F-actin interactions in the maintenance of vesicle clusters. Considering that palmitoylation occurs in another part (domain C) of Syn1 but not the IDR, these two models do not necessarily exclude each other; rather, together they might reflect different aspects of Syn1 and the regulatory mechanisms of SVs clustering.

Syn1 palmitoylation has not yet been reported, though Syn1 is one of the most abundant proteins in the presynaptic terminus [26]. A related palm-proteomics study reported that Syn1 was not observed in synaptosomes prepared from cultured embryonic cortical neurons [27]. This discrepancy might come from the fact that Syn1 is weakly expressed at the embryonic stage [28].

Syn1 contains several domains (A-E) in its protein sequence, and studies of these truncated domains have found that each domain has distinct functions [20, 29, 30], e.g., domain A solely controls SVs release in a phosphorylation-dependent manner at S9, while domain C may be involved in regulation of binding with F-actin and controlling the size of SVs pool and release kinetics [6, 31, 32]. One important question is how these individual domains coordinate with each other and exert physiological functions. Here, we describe how phospho-Syn1 (S9, located in domain A) negatively regulates its own palmitoylation (C223, 360, and 370, located in domain C) (Fig. 5A–D), but not the reverse (Fig. 5L–Q), suggesting that disjoined domains within Syn1 crosstalk with one other to fulfill its molecular functions. Domain C is well preserved among different isoforms of Syn1 (Syn1-3), and these isoforms could form both homo- and heterodimers or tetramers (mediated by D290, W335 and K336) via domain C. Whether palmitoylation occurs in domain C of other isoforms of Syn1 and if palmitoylated domain C might have an impact on the formation of Syn-complexes and the binding of actin require further study.

It was demonstrated that PKA-triggered Syn1 phosphorylation causes the diffusion of SVs to the periphery region [17, 33, 34], and a similar phenomenon is observed in neurons expressing Syn1-3CA (Fig. 2A–I), implying that Syn1 phosphorylation and palmitoylation might function in the same signaling cascade. However, they differ mechanically: phospho-Syn1 alters its binding with both SVs [20, 32] and F-actin [4, 21, 22], while Syn1 depalmitoylation only interferes with its binding with F-actin but not SVs (Fig. 4A–M). Further investigation determined that Syn1 phosphorylation (FSK-activated) triggered SVs release rely on the depalmitoylation process of Syn1, potentially mediated by ABHD17a, as inhibiting such process by either PalmB or the depletion of ABHD17a suppresses SVs redistribution in FSK-treated neurons (Fig. 6C, D and K, L). These observations led us to speculate that the rapid release of the SVs pool at the presynapse might require sequential actions: 1) the detaching of Syn1 from SVs triggered by phospho-Syn1 and 2) the detaching of Syn1 from F-actin triggered by depalmitoylated-Syn1.

It is clear that actin is abundantly expressed in presynaptic terminals and dynamically responds to neuronal activities [35, 36], yet, the precise roles of actin within the presynaptic domain are yet unclear. One study demonstrated that disrupting actin dynamicity by short-term latrunculin A treatment (seconds-minutes) does not affect SVs clustering [35], while other studies showed that long-term latrunculin A treatment (hours) could abolish most of the polymerized F-actin and reduce mEPSC frequency [37, 38]. The discrepancy within these observations might result from varied levels of damage to actin networks caused by different durations of latrunculin A treatment. Combined with the finding that palm-Syn1 facilitates its binding with actin, and vice versa (Fig. 4), we speculate that actin may serve as a scaffold matrix within the presynaptic locus to recruit palm-Syn1 and thus affect SVs dynamicity.

The ablation of Syn1 leads to severely dispersed SVs (enlarged distance between SVs) and considerably reduced total number of SVs within the presynapse [39, 40], as phenocopied in neurons expressing Syn1-3CA (Fig. 2E–I) or hippocampal neurons from ZDHHC5-KO mice when palm-Syn1 is downregulated (Fig. 3J–N), supporting the hypothesis that Syn1 and its palmitoylation are involved in SVs clustering. Combined with the finding that palm-Syn1 facilitates its interaction with F-actin, and vice versa (Fig. 4C–N), we reasoned that the phenotype of dispersed SVs in vivo might be caused by the impaired interaction between Syn1 and F-actin in either Syn1-KO mice or ZDHHC5-KO mice (Fig. 3J–N); specifically, Syn1-associated SVs fail to anchor on F-actin to form dense SVs clusters, indicated by loosely compacted SVs and a reduced number of total SVs at the presynapse (Fig. 2E–K and Fig. 3J–N). Palmitoylation facilitates Syn1 to interact with F-actin: it is possible that palmitoylation enhances the hydrophobicity of domain C and thus causes an intramolecular switch to remodel its binding to F-actin with higher affinity.

Future research into this topic should take several directions. First, considering that the deletion of ZDHHC5 partially alleviates (40%) palm-Syn1 and the removal of ABHD17a strongly augments palm-Syn1 in vivo, other palmitoylation-related enzymes might be also involved, and should be investigated. ZDHHC15 and ZDHHC19 are capable of catalyzing Syn1 palmitoylation in vitro (Fig. S3A, B), yet a recent study reported that DHHC19 is mainly expressed in the mouse testis but not in the brain [41], which is in line with our finding (Fig. S3C). Although DHHC15 is important for dendrite outgrowth and spine maturation in embryonic neurons, it is highly expressed in earlier developmental stages (E17-P10) but significantly reduced in the adult brain (P90) [42]. Interestingly, DHHC5 increases its expression in mouse hippocampus together with Syn1 at different developmental stages (Fig. S6E). Considering that neuronal activity (SVs clustering and release) is a predominant molecular event in adult nervous system, DHHC5 is an appropriate candidate for Syn1 palmitoylation. For depalmitoylation, PPT1 was shown to reduce palm-Syn1 in vitro (Fig. S8A–C) and is involved in SV recycling at the presynapse [43]; however, the loss of PPT1 [19] does not detectably alter palm-Syn1 in vivo (Fig. S8H). Interestingly, ABHD17a/b/c show redundant functions [44, 45] catalyzing Syn1 depalmitoylation in vitro (Fig. S9D, E). Our results showed that ABHD17c, but not ABHD17b, is highly expressed in cultured hippocampal neurons (Fig. S9E). The deletion of ABHD17a significantly augments palm-Syn1 in mice brains (Fig. 6G, H), indicating that either ABHD17c is not highly expressed in protein level, or that it has a different functional locus than that of the Syn1 in neurons. Second, although we showed that ZDHHC5/ABHD17a-regulated Syn1 palmitoylation is important for SVs clustering and redistribution, other potential substrates of ZDHHC5/ABHD17a at the presynaptic locus might also contribute to SVs dynamicity, e.g., SVs docking (Fig. 3M), which warrants further investigations.

Collectively, we showed that Syn1 is dynamically palmitoylated by ZDHHC5 and ABHD17a in vivo, and established that FSK-induced Syn1 phosphorylation downregulates palm-Syn1, with the latter alleviating its binding with F-actin, which results in SVs redistribution and reduced number of total SVs at the presynapse. Further, as phospho-Syn1 wanes, Syn1 is palmitoylated by ZDHHC5 and hence binds F-actin and SVs for clustering (Fig. 7A). Our findings provide new insights and offer a refined model for SVs dynamics within presynaptic domains in neurons.

## Materials and methods

### Animals and animal care

Wild-type C57BL6 (B6) mice were purchased from Beijing Vital River Laboratory Animal Technology Co., Ltd. All mice were kept at SPF environment and housed in colony cages at 25 °C with a 12-h light, 12-h dark cycle and free access to food and water. All animal procedures were performed according to guidelines approved (2019S002) by the ethic committee on animal care at Xinxiang Medical University. Adult male mice were randomly allocated and used for all experiments.

### Generation of synapsin1-KO mice

Fertilized B6 eggs were harvested and injected with microinjection system. In brief, Cas9 mRNA and sgRNA (GTGGACAGTTGCGTCTGAATAGG, GATTTTGAAATATTCGTATAAGG) were generated by using in-vitro transcript (IVT) kits, all components were mixed well and injected into the cytoplasm of fertilized eggs. Handled eggs were cultured to two-cell stage and transferred into ICR foster mice. 20 days later, F0 mice were born and genomic DNA was isolated. The following primer pairs: F: GGAGTGTCTCTTAATTTCATGTCCC and R1: TGCTTCTTGGTCATATTTGTGCAG R2: GCATGGAGGTCTGTAAATGGCTAAG were used for genotyping purpose. The amplicons of WT and knockout alleles are 498 bp and 630 bp, respectively (Fig. S2).

### Generation of zdhhc5-KO mice

Similar procedures were performed as above. The following sgRNA (TTGTAAGCTGATTGATGTACAGG, GCTTAATTAGTGAAGGCATCAGG) were designed and used. For genotyping, the following primer pairs were used: F: CTAAAGGTACTGATAGTTGGTTCTG and R1: CTTTCTTAACCCAAACCATCCAGT, R2: CTAAAGTTTGGCATGGTGCTACA. The amplicons of WT and knockout alleles are 729 bp and 488 bp, respectively (Fig. S5).

### Generation of ABHD17a-KO mice

Similar procedures were performed as in Syn1-KO mice. The following sgRNAs (sg1-GGCTCGCCTTGGACCGCGATGGG, sg2-TCACATGCAGTCCCGGCAGGAGG and sg3-GAGCCATGTTCAGGCGTCAGAGG) were produced and used. For genotyping, the following primer pairs: F: CATAGAATGAGTTCTGCCACAGAG and R1: CTTTGGACCAGGAATCTTCGCATC, R2: TGAAAGCAAGAACATGAAAGTGGAG were used. The amplicons of WT and knockout alleles are 811 bp and 556 bp, respectively (Fig. S10).

### Deleting ZDHHC5 in HEK-293T cell

HEK-293T (CRL-11268) was purchased from ATCC and cultured in Dulbecco’s modified Eagle’s medium (DMEM, Hyclone) supplemented with 10% fetal bovine serum (FBS, GIBCO, Thermo Fisher Scientific) and 1% Pen/Strep (Hyclone) at 37 °C in an atmosphere of 5% CO_2_. For targeting human ZDHHC5, two sgRNAs (GCATCCAGGTGCGCATGAAATGG, ATGAAGTCTTACCCCACCCAGGG) were designed and cloned into pX458 vector with GFP to enable cell sorting, which were then transfected into HEK-293T cells using Lipofectamine 3000 (Invitrogen) according to the manufacturer’s instructions. After 48 h, single cell was sorted by BD FACS Aria^TM^ Fusion and then cultured until the formation of colonies. Paired primers (F: ATTTCCGAGCTCCCCTT, R: CTGCCTCTCATGCCATGTCA) were used for screening the positive clone and then confirmed by sanger sequencing (Fig. S4).

### Deleting ABHD17a in HEK-293T cell

Similarly, for targeting human ABHD17a in HEK-293T cell, two sgRNAs (GAGAAGAGGACCGTGTACCTGGG, GAGCAGCTTCTACATTGGCCTGG) were designed to delete exon 4 in ABHD17a (Fig. S8A). For genotyping, the the following primers: F: GGCTGTCACTACGCATCCT, R: TGTCGGCATAGAGGTTCCTC were used to screen for positive clone, which were then sequenced for verification (Fig. S9).

### Plasmids

The cDNA sequences of mouse syn1a, zdhhc5, apt1, apt2, ppt1, ppt2, and ABHD17a were cloned into pCMV3 vector with tags of either his, flag or GFP on the C-terminus. The pCMV3-YFP^n^-syn1a and pCMV3-YFP^c^-β-actin plasmids carry either the N-terminus of YFP (1–174 aa) or the C-terminus of YFP (175–238 aa) were fused with either Syn1 or Beta-actin for BiFC assay. HA-tagged DHHC-PATs were from the lab of Dr. Fukata. For lentiviral infection, the cDNA of mouse syn1a and its mutant (Cys 223/360/370 A) were cloned into pGV218 vector and the virus was packaged in HEK-293T cells, prepared by Gene Incorporation. DHHC5 shRNA (in vector GV298) targeting mouse (5’-CCTCAGATGATTCCAAGAGAT-3’) was packaged in HEK-293T cells by Gene Incorporation.

### Drug treatments

Cultured cells were incubated with the following drugs for different aims. 2-Bromopalmitate (2-BP, Sigma-Aldrich, Cat # 238422) was used at 50 μM for various time periods, Forskolin (FSK, MCE, Cat # HY-15371) and Palmostatin B (Palm B, Calbiochem, Cat # 178501) were used with the concentrations of 10 μM for either 0.5 h or 1 h.

### Purification of Syn1

Syn1-Flag was expressed in HEK-293T cells and harvested with lysis buffer (20 mM Tris pH 7.5, 150 mM NaCl, 1% Triton X-100) containing protease inhibitors (Roche). Total protein was clarified by centrifugation at 12,000 × *g* for 30 min at 4 °C. The Anti-DYKDDDDK Affinity resin (Sino Biological, Cat # 101274-MM13-RN) was equilibrated in equilibrating buffer (PBS 10 mM, pH 7.4) 3 times and then incubated with the pre-cleared lysates at 4 °C with rotation for overnight to pull down Syn1-flag. For harvest, the resin was pelleted by centrifugation and washed in equilibrating buffer 4 times and eluted in eluting buffer (100 mM Glycine, 10 mM NaCl, pH 3.0). The eluted proteins were then validated by mass spectrometric analysis. For the following binding assay, about 1:50 volume of 1 M HEPES (PH 9.5) was added to adjust the pH to 7.4.

### Purification of synaptic vesicles from mice brains

Synaptic vesicles were purified from mouse brain as described [46]. Briefly, mice cerebral cortices were homogenized with glass-Teflon homogenizer in ice-cold buffered sucrose (4 mM HEPES, 320 mM Sucrose, pH 7.3) and centrifuged at 800 × *g* for 10 min at 4 °C. The supernatant was centrifuged at 9200 × *g* for 15 min at 4 °C and the subsequent supernatant was discarded. The pellet was resuspended with 40 ml of buffered sucrose and then centrifuged at 10,200 × *g* for 15 min at 4 °C. Again, the supernatant was discarded and the pellet was resuspended in 6.5 ml buffered sucrose. 58.5 ml of ice-cold water was then added and the suspension was homogenized with a glass-Teflon homogenizer briefly at maximum speed. 1 M HEPES-NaOH (pH 7.4) was added to the suspension to achieve a final concentration of 3 mM HEPES and incubated on ice for 30 min, then centrifuged at 25,000 × *g* for 20 min at 4 °C. The supernatant was further centrifuged at 165,000 × *g* for 2 h. The pellets were resuspended with 40 mM sucrose (1.2 ml) and layered on top of a continuous sucrose gradient, generated by 5 ml of 800 mM sucrose and 5.8 ml of 50 mM sucrose. Sucrose gradient centrifugation was performed for 5 h at 65,000 × *g* and the fractions with high turbidity were pooled and further centrifuged for 2 h at 175,000 × *g* at 4 °C. The pellets were resuspended at a protein concentration of 2–3 mg/ml in 0.3 M glycine, 5 mM HEPES-NaOH (pH 7.4), 0.02% NaN_3_ and used within 3 days of preparation.

### Affinity-binding assay

Purified synaptic vesicles (SVs) were depleted of endogenous synapsins by dilution in 0.2 M NaCl and centrifuged at 200,000 × *g* for 2 h after 2 h incubation at 0 °C [46]. After centrifugation, SVs were resuspended in 0.3 M glycine, 5 mM HEPES-NaOH, pH 7.4 at a protein concentration of 1.5–2 mg/ml. The binding of Syn1 to synapsin-depleted SVs was carried out using a high-speed sedimentation assay. Briefly, SVs (5–10 μg total protein) were incubated for 1 h at 0 °C with 15 nM Syn1-WT-flag or its mutants in a buffer containing 220 mM glycine, 30 mM NaCl, 5 mM Tris/HCl, 4 mM HEPES (pH 7.4), 0.5 mM CaCl2, 0.22 mM NaN3 and 100 μg/ml of BSA. After incubation, samples were placed on top of 5% sucrose (wt/vol) in glycine buffer and subjected to ultracentrifugation at 200,000 × *g* for 1 h to separate unbound Syn1. The supernatants were carefully aspirated and the pellets were resuspended in 80 μl of “spot buffer” containing 160 mM NaCl, 10 mM NaPO4 (pH 7.4), and 1.4% (vol/vol) Triton X-100. The resuspended pellets were subjected for SDS-PAGE and western blotting analysis.

### F-actin-binding assay

As described [4, 47], G-actin (5 μM, Sigma-Aldrich, Cat # A2522) was polymerized for 1 h at room temperature in 12.5 mM NaCl, 0.6 mM ATP, 100 mM KCl, 1.2 mM MgCl2, 0.5 mM CaCl2, 1.5 mM 2-mercaptoethanol, 6 mM HEPES, 8 mM Tris (pH 7.4) with the presence of Syn1-WT (0.25 μM) or its mutants. Samples were centrifuged at 200,000 × *g* for 30 min and the actin pellets were solubilized in SDS-sample buffer and subjected to SDS-PAGE. The amount of Syn1 bound to F-actin was determined by western blotting. For hydroxylamine (HA) treatment, purified Syn1 was incubated with same volume of 1 M HA (PH7.5) at 37 degree for 2 h, and then HA was removed by ultrafiltration.

### Determining Syn1 palmitoylation by mass-spectrometry

As previously described [8, 48], purified Syn1-flag (30 µg) were digested using FASP and the disulfide bonds were broken and blocked using 2 mM TCEP and 10 mM iodoacetamide. Then proteins were transferred to 10 K filter, and cleaned sequentially using 8 M urea and 50 mM Tris-Hcl (pH 6.8) at 13,000 × *g*, 20 °C. GluC (P8100S, BioLabs) was added to filter at 1:50 (mass/mass) in 1x reaction buffer and proteins were digested at 37 °C for 16 h. Peptides were collected, lyophilized and stored at −80 °C until use. Raw files were acquired with data-dependent acquisition mode using Orbitrap Fusion Lumos (San Jose, Thermo Fisher). Peptide mixture were separated on EasyNano LC1000 system (San Jose, Thermo Fisher) using both C18 and C4 column at a flowrate of 600 nl/min. To better identify modified amino acid sites, each precursor ion was fragmented with both HCD and EThcD. Raw files were searched against target protein sequence using Byonic v2.16.11 (Protein Metrics). An automatic score cut was used to remove low-score peptides. A manual check was applied to further filter high-confident palmitoylated cysteine sites. Modified peptides only with continuous b and y product ions can be considered as a high-confident modified site.

### Primary neuron culture and transfection

Mice hippocampus were dissected out from P0-P2 pups and digested by 2.5% Trypsin (GIBCO, Thermo Fisher Scientific) for 15 min at 37 °C with gentle agitation. Tissues were then washed 3 times with DMEM (supplemented with 5% FBS, 1% Glutamax, 1% sodium pyruvate and 1% Pen/Strep) and triturated with a fire-polished pasteur pipette until the suspension is homogenous and filtered by falcon filter (BD Biosciences). Neurons were seeded (0.2–0.3 × 10^6^ cells/well) in 12-well plate on glass coverslips coated with poly-d-lysine for immunofluorescence or in 6-well plate (0.5–0.6 × 10^6^ cells/well) coated with poly-D-lysine. Next morning, DMEM was replaced by Neurobasal medium (NBM, GIBCO, Thermo Fisher Scientific) supplemented with 2% B27, 1% Glutamax, and 1% Pen/Strep for long-term maintenance. Lipofectamine 2000 (Invitrogen) was used for transient transfection according to manufacturer’s instructions at DIV 8-9, alternatively, neurons were infected with lentiviruses expressing corresponding Syn1 proteins.

### Acyl-RAC assay

*Acy*l-RAC assays were performed as described previously [8]. Total amount of around 1 mg protein were used typically. In principal, the free thiol groups were blocked by N-ethylmaleimide (NEM, Sigma-Aldrich, Cat # 04259) at 50 °C for 1 h in blocking buffer (100 mM HEPES, 1.0 mM EDTA, 2.5% SDS, 50 mM NEM, pH 7.5). Followed by three times protein precipitation using four volumes of cold acetone at −20 °C for 30 min and centrifugation at 10,000 × *g* for 10 min. The pellet was washed three times with 70% acetone and resuspended in 1.2 ml of binding buffer (100 mM HEPES, 1.0 mM EDTA, 1% SDS, pH 7.5) containing protease inhibitor at the final time. The sample was divided into two parts and treated with either 2 M NH_2_OH (+HA) or 2 M NaCl (–HA). Then, 50 μl of prewashed thiopropyl Sepharose 6B beads (GE Healthcare, Cat # 17-0420-01) was added, and the reaction was carried out on a rotator at room temperature for 4 h. After washing, proteins were eluted with 50 μl of Laemmli loading buffer (2.1% SDS, 66 mM Tris, 26% glycerol w/v, 50 mM DTT, pH 7.5) and subjected for SDS-PAGE analysis.

### Metabolic labeling of palmitate and click chemistry

As described [14], cells were incubated with 50 μM 17-ODYA (Cayman Chemical, Cat # 90270) overnight at 37 °C. To facilitate dissolving 17-ODYA in the medium, 37.5 μl 20 mM 17-ODYA stock in DMSO was premixed with 75 μl 10% fatty acid free BSA (Sigma-Aldrich), added to 15 ml medium, vortexed, and then take 7 ml per plate (10 cm dish). For harvesting, cells were washed with PBS and lysed in lysis buffer [50 mM Tris-HCl [pH 7.4], 150 mM NaCl, 0.1% Triton X-100, 0.1% SDS] supplemented with protease inhibitor cocktail. The cell lysate was clarified at 13,400 × *g* for 10 min at 4 °C and the supernatant was subjected to click reaction. An aliquot (94 μl) of each cleared lysate were transferred to fresh tubes and the following reagents added individually to perform the Cu-catalyzed click reaction: 1 μl of 10 mM biotin-azide stock in DMSO, 2 μl of 50 mM TCEP, PH 7.5 stock in H_2_O, 1 μl of 10 mM TBTA stock in DMSO, 2 μl of 50 mM CuSO_4_ stock in H_2_O. Reaction mixtures were incubated for 1 h at room temperature (RT) with intermittent mixing, and then 2 μl of 0.5 M EDTA was added to terminate the reaction. Then samples were precipitated with chloroform-methanol, washed twice with cold methanol to quench the unreacted biotin. Biotinylated (palmitoylated) proteins were then affinity isolated using streptavidin beads by incubation at 4 °C for 4 h. The beads were washed 3 times with PBS containing 0.5% Triton X-100 and treated with sample buffer for western blot analysis.

### Reverse transcription and real-time PCR

Total RNA was extracted using the RNA Easy Fast Tissue/Cell Kit (Tiangen, Cat # DP451) according to the manufacturer’s protocol. Total RNA (1 µg) was reverse-transcribed into complementary DNA (cDNA) using PrimeScript™ RT Master Mix (Takara, Cat # RR036Q). Quantitative real-time PCR was performed using TB Green® Premix Ex Taq™ II (Takara, Cat # RR820A) according to the manufacturer’s protocol on CobasTM z 480 (Roche). The sequences of the primer used are listed in supplemental Table 1. GAPDH was used as an internal control and the relative quantity of each detected genes were normalized to the housekeeping gene (ΔCt), which was then normalized again with that of the control group (ΔΔCt) and calculated by 2^–ΔΔCt^. Each experiment was performed in triplicate and values were averaged.

### Immunofluorescence staining and imaging

In general, cultured cells were fixed with 4% paraformaldehyde for 20 min at room temperature, permeabilized with 0.1% Triton X-100 in PBS for 5 min and blocked with 3% BSA in PBS for 1 h. Following primary antibodies were used: anti-Syn1 (1:500, abcam, ab64581), anti-Flag M2 antibody (1:1000, Sigma, F1804), anti-Syp (1:500, abcam, ab32127), anti-VAMP2 (1:500, abcam, 181754), and anti-Bassoon (1:250, abcam, ab82958), coupled with Alexa Fluor 488 goat anti-rabbit or Alexa Fluor 647 goat anti-mouse secondary antibody (1:1000, Thermo Fisher Scientific). After washing, cells were mounted with Fluoromount-G (Electron Microscopy Sciences) and images were acquired using Stimulated Emission Depletion microscopy (Leica TCS SP8 STED). The fluorescence intensity and colocalization rates were measured by LAS X (version: 3.3.0.16799), a software associated with the microscopy.

### Western blotting and antibodies

*Samples were separated in standard SDS-PAGE gels and transferred to Immobilon-P PVDF me*mbrane (EMD Millipore), blocked in 5% skimmed milk in TBS containing 0.1% Tween-20 for 1 h. Primary antibodies were incubated at 4 degree overnight, after washing, a suitable horseradish peroxidase (HRP) labeled secondary antibody was added for detecting signals with an ECL kit (Tanon). The following antibodies were used: anti-Syn1 (1:1000, abcam, ab64581), anti-Syn1 phospho S9 (1:1000, abcam, ab76260), anti-Syn1 phospho S553 (1:1000, abcam, ab32532), anti-Syn1 phospho S603 (1:1000, abcam, ab13879), anti-Syp (1:20,000, abcam, ab32127), anti-pan actin (1:1,000, abcam, ab14128-C4), anti-β-actin (1:1000, Sigma, A2228), anti-HA (1:1000, CST, #2367), anti-His (1:1000, abcam, ab18184), Mouse anti DDDDK mAb (1:5,000, ABclonal, AE024), Goat Anti-Rabbit IgG (H + L), HRP Conjugate (Protein Biotechnologies, Cat# PMS302, 1:5000) and Goat Anti-Mouse IgG (H + L), HRP Conjugate (Protein Biotechnologies, Cat# PMS301, 1:5000).

### Sample preparation and imaging for electron microscopy

Cultured neurons were fixed at DIV14-15 with 2.5% glutaraldehyde in 0.1 M PB for 30 min. Then samples were shipped on ice to Peking University for further processing. After washing with 0.1 M PB four times for 8 min each, cells were post-fixed with 1% OsO_4_ in 0.1 M PB containing 0.8% K_4_Fe(CN)_6_ for 1 h in the dark. fully rinsed with ultrapure water, stained in 1% aqueous uranyl acetate overnight at 4 °C. After washing thoroughly in ultrapure water, cells were dehydrated in graded series of ethanol, infiltrated, embedded in epoxy resin (EMbed 812, Electron Microscopy Sciences), polymerized at 65 °C for 24 h. Resin blocks were trimmed and sectioned on an ultramicrotome (UC7, Leica Microsystem) equipped with a diamond knife (ultra 35°, Diatome, Switzerland). Ultra-thin sections (70 nm) were collected on single-slot grids coated with formvar film, contrasted with uranyl acetate and lead citrate and examined with a transmission electron microscope (Tecnai G2 Spirit BioTWIN, FEI) operating at 120 kV. Digital images were obtained with Gatan 832 CCD camera (Gatan, Pleasanton, CA). At least 40 images were acquired at×12,500 magnification and synaptic profiles, e.g., mean nearest neighbor distance (MNND) was quantified by ImageJ.

Similarly, anesthetized mice were perfused with saline followed by perfusion with freshly prepared fixative containing 4% paraformaldehyde and 2.5% glutaraldehyde supplied with 0.1 M sucrose in 0.1 M phosphate buffer (PB), pH 7.4. Targeted brain region was chopped into 1 mm3 blocks, kept for 2 h at room temperature, then shipped on ice to Peking University for further processing. The following sample preparation procedure were carried out as mentioned above.

### FM4-64 uptake experiment

As described [17], cultured neurons (DIV14–15) expressing Syn1-WT-GFP or Syn1-3CA-GFP were stimulated for 1 min with 55 mM KCl, in the presence of 10 μM FM4-64 FX (Thermo Fisher Scientific, Cat # F34653) and 1 μM TTX (Cayman, Cat # 14964) in Krebs’–Ringer’s–HEPES solution (KRH, 130 mM NaCl, 5 mM KCl, 1.2 mM MgSO_4,_ 1.2 mM KH_2_PO_4_, 2 mM CaCl_2_, 6 mM glucose, and 25 mM HEPES, pH 7.4). Followed by two times complete medium substitutions, neurons were perfused for 10 min with warmed KRH (37 °C) supplemented with 1 μM TTX and 10 μM CNQX (MCE, Cat # HY-15066) and fixed with 4% paraformaldehyde for 10 min on ice. After 3 times washing with ice-cold HBSS, the coverslip was mounted for imaging. FM4-64 fluorescence was measured on digital images within an area of 3 × 3 pixels at the center of synapses expressing Syn1-WT-GFP or Syn1-3CA-GFP as indicated.

### Statistical analysis

*Basi*c descriptive data are presented as means ± standard errors of means (s.e.m). Primary data processing and organization were performed in Microsoft Excel (2010). Statistical analyses were performed using GraphPad Prism 7.0 software (GraphPad, USA). Statistical significance for two groups was determined by unpaired two-tailed Student’s t-test. In the case of unequal variance between the two groups, the unpaired Welch’s t-test was used. For determining differences between more than two groups, one-way ANOVA was applied and followed by Dunnett T3’s post hoc test (data with unequal variance) or by Tukey’s post hoc test (data with equal variance) as indicated. Significance was defined as: n.s., not significant, *p < 0.05, **p < 0.01, ***p < 0.001, and ****p < 0.0001. None of the samples were excluded from the statistical analysis. Sample sizes referred to the general application of the field and were not statistically predicted.

## Supplementary information

Supplemental figures and table

Supplemental figure legends

Supplementary file 1

Reproducibility checklist

## Data Availability

The published article includes all data sets generated/analyzed for this study. Additional data are available from the corresponding author on reasonable request.
