# Supplementary material for: Crosstalk of Synapsin1 palmitoylation and phosphorylation controls the dynamicity of synaptic vesicles in neurons
Source: Cell Death Dis. 2022 Sep 12;13(9):786. doi: 10.1038/s41419-022-05235-4 (PMC9468182; doi:10.1038/s41419-022-05235-4)
Supplement: Supplementary file 1 — Supplemental figures and table [file 41419_2022_5235_MOESM1_ESM.pdf]

FIG. S1

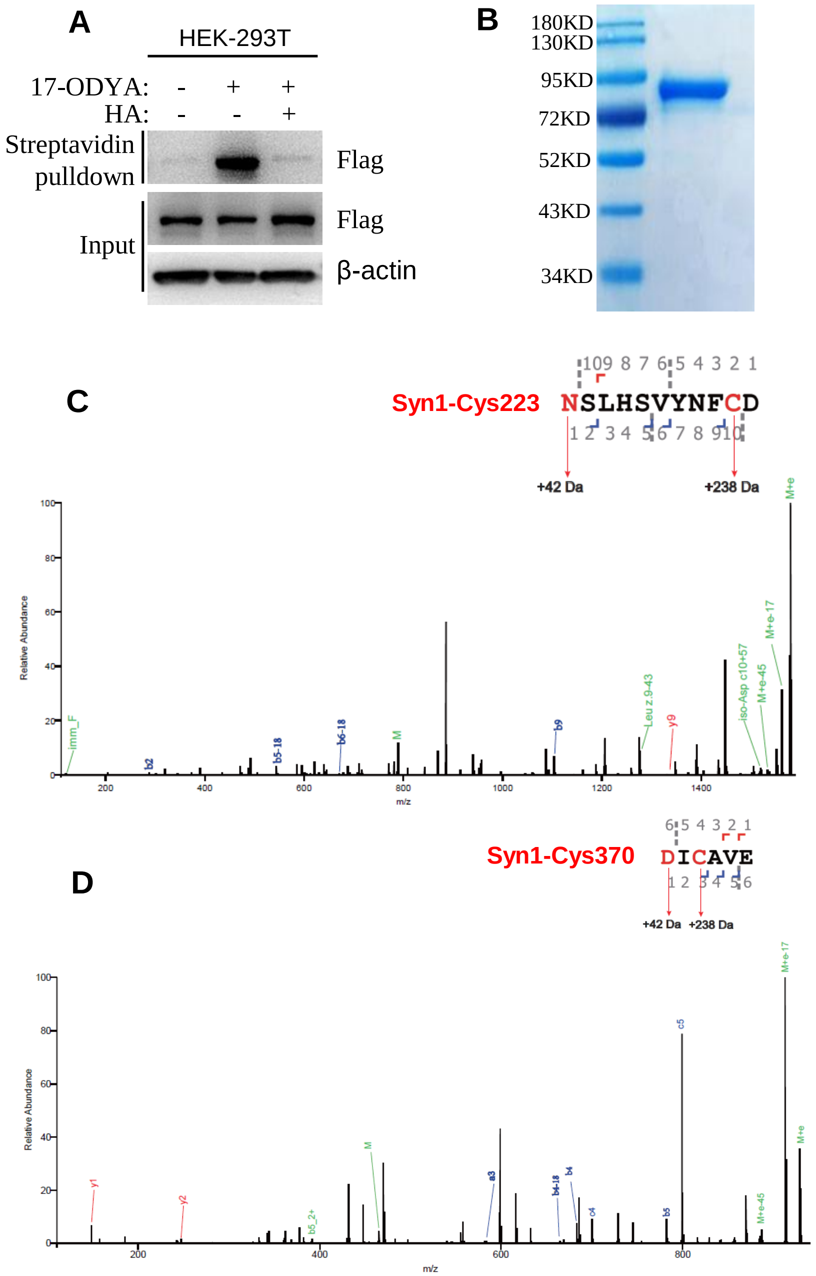

A
HEK-293T
17-ODYA: - + +
HA: - - +
Streptavidin pulldown
Flag
Input
Flag
β-actin
B
180KD
130KD
95KD
72KD
52KD
43KD
34KD
C
Syn1-Cys223
NSLHSVYNFCD
+42 Da
+238 Da
Relative Abundance
m/z
D
Syn1-Cys370
DICAVE
+42 Da
+238 Da
Relative Abundance
m/z

FIG. S2

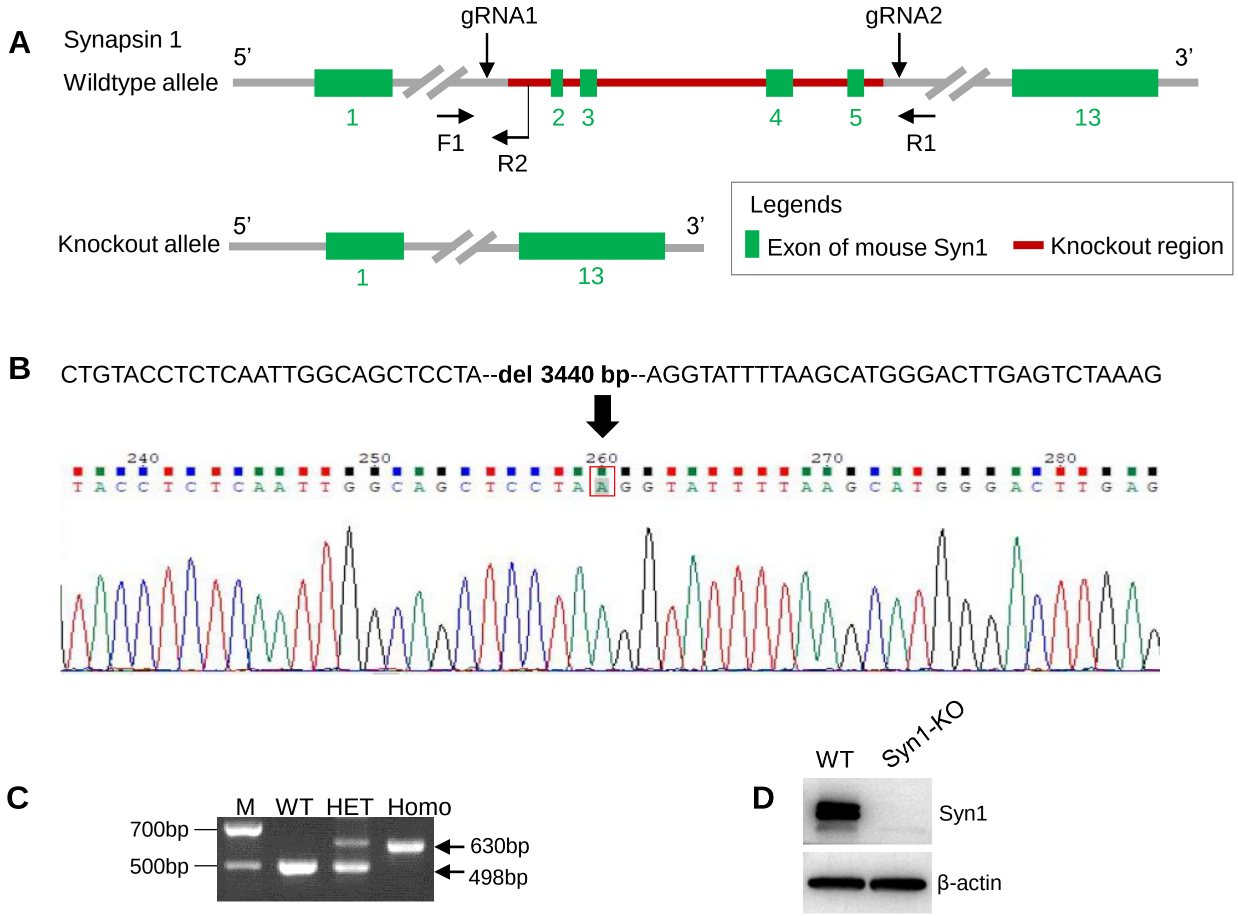
A
Synapsin 1
Wildtype allele
gRNA1
gRNA2
5'
3'
1
2 3
4
5
13
F1
R2
R1
Knockout allele
5'
3'
1
13
Legends
Exon of mouse Syn1
Knockout region
B
CTGTACCTCTCAATTGGCAGCTCCTA--**del 3440 bp**--AGGTATTTTAAGCATGGGACTTGAGTCTAAAG
240
250
260
270
280
T A C C T C T C A A T T G G C A G C T C C T A A G G T A T T T T A A G C A T G G G A C T T G A G
C
M WT HET Homo
700bp
500bp
630bp
498bp
D
WT
Syn1-KO
Syn1
β-actin

FIG. S3

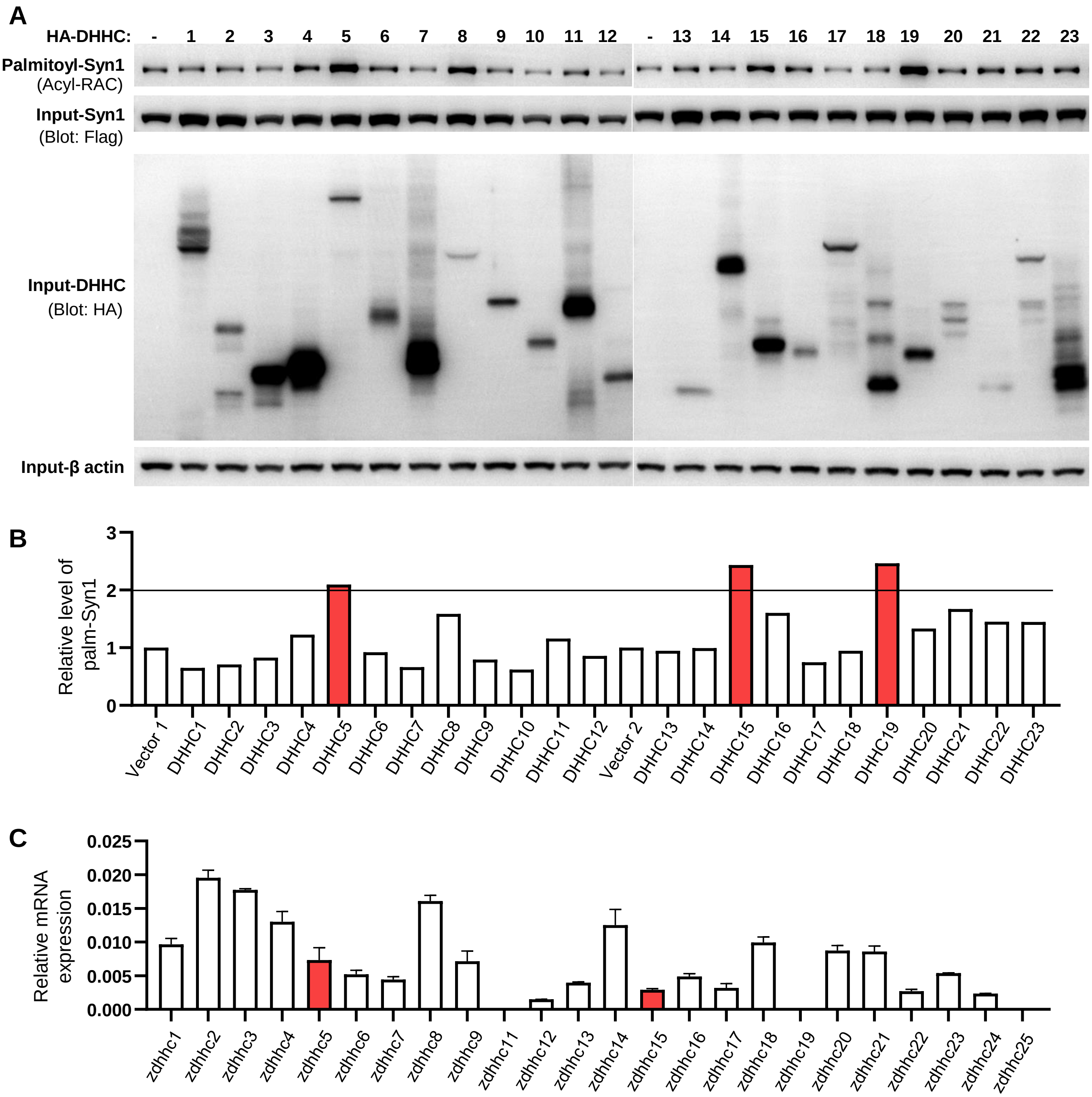
A
HA-DHHC: - 1 2 3 4 5 6 7 8 9 10 11 12 - 13 14 15 16 17 18 19 20 21 22 23
Palmitoyl-Syn1
(Acyl-RAC)
Input-Syn1
(Blot: Flag)
Input-DHHC
(Blot: HA)
Input-β actin
B
Relative level of palm-Syn1
0
1
2
3
Vector 1
DHHC1
DHHC2
DHHC3
DHHC4
DHHC5
DHHC6
DHHC7
DHHC8
DHHC9
DHHC10
DHHC11
DHHC12
Vector 2
DHHC13
DHHC14
DHHC15
DHHC16
DHHC17
DHHC18
DHHC19
DHHC20
DHHC21
DHHC22
DHHC23
C
Relative mRNA expression
0.000
0.005
0.010
0.015
0.020
0.025
zdhhc1
zdhhc2
zdhhc3
zdhhc4
zdhhc5
zdhhc6
zdhhc7
zdhhc8
zdhhc9
zdhhc11
zdhhc12
zdhhc13
zdhhc14
zdhhc15
zdhhc16
zdhhc17
zdhhc18
zdhhc19
zdhhc20
zdhhc21
zdhhc22
zdhhc23
zdhhc24
zdhhc25

FIG. S4

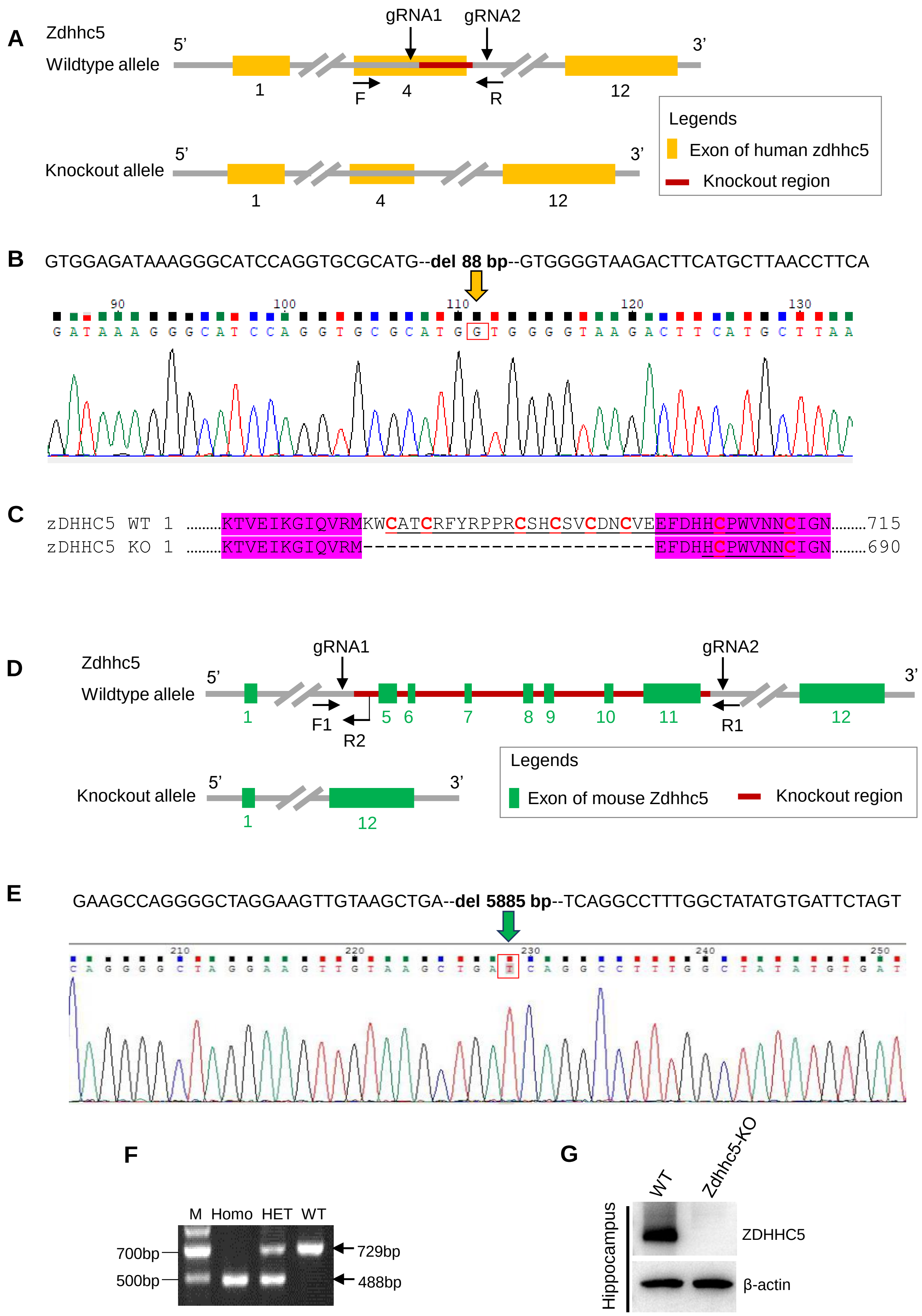

A
Zdhhc5
Wildtype allele
gRNA1
gRNA2
5’
3’
1
F
4
R
12
Knockout allele
5’
3’
1
4
12
Legends
Exon of human zdhhc5
Knockout region
B
GTGGAGATAAAGGGCATCCAGGTGCGCATG--del 88 bp--GTGGGGTAAGACTTCATGCTTAACCTTCA
90
100
110
120
130
C
zDHHC5 WT 1 .........KTVEIKGIQVRMKWCATCRFYRPPRCSHCSVCDNCVEEFDHHCPWVNNCIGN.........715
zDHHC5 KO 1 .........KTVEIKGIQVRM-------------------------EFDHHCPWVNNCIGN.........690
D
Zdhhc5
Wildtype allele
gRNA1
gRNA2
5’
3’
1
F1
R2
5 6
7
8 9
10
11
R1
12
Knockout allele
5’
3’
1
12
Legends
Exon of mouse Zdhhc5
Knockout region
E
GAAGCCAGGGGCTAGGAAGTTGTAAGCTGA--del 5885 bp--TCAGGCCTTTGGCTATATGTGATTCTAGT
210
220
230
240
250
F
M Homo HET WT
700bp
500bp
729bp
488bp
G
WT
Zdhhc5-KO
Hippocampus
ZDHHC5
β-actin

FIG. S5

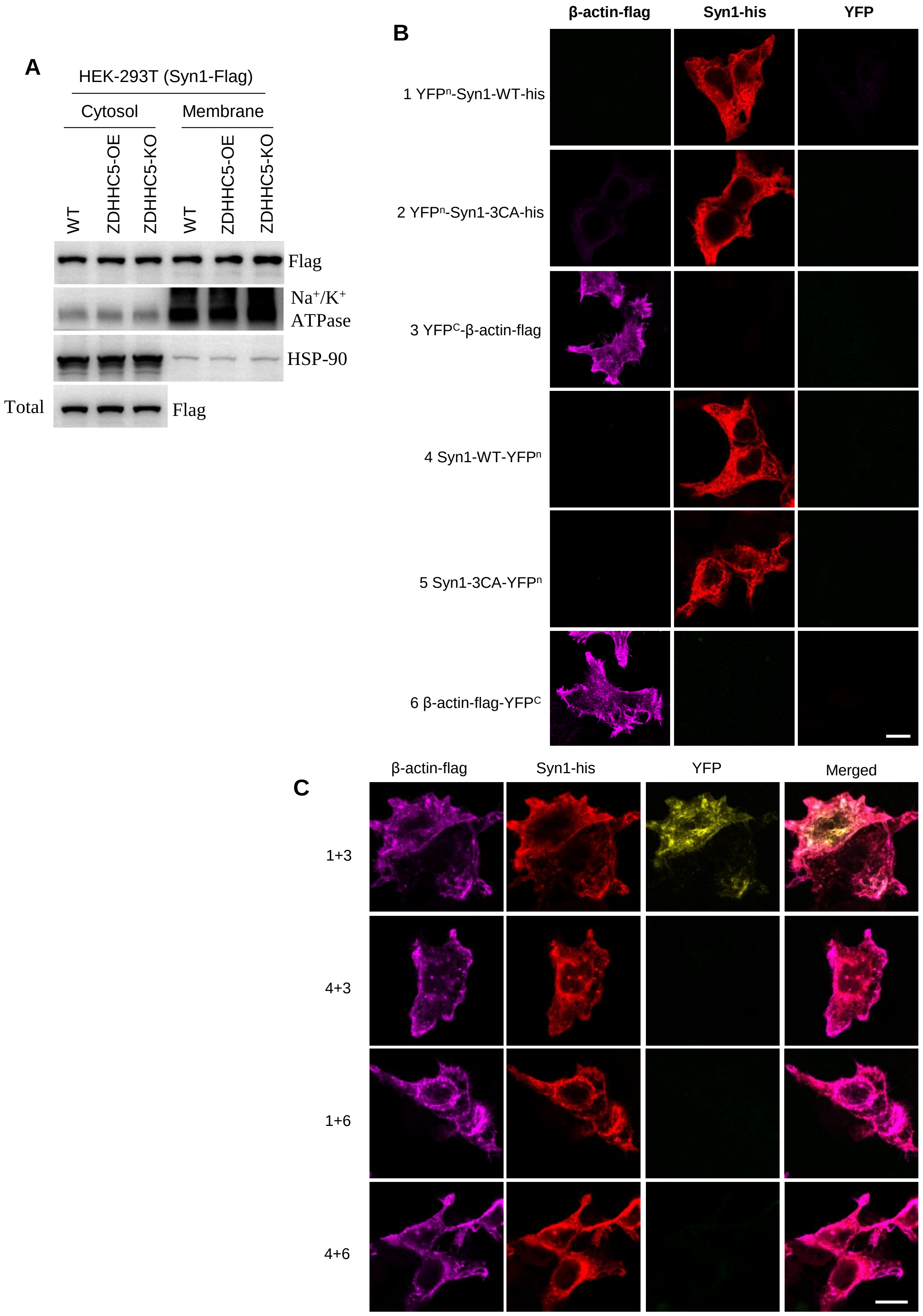
A
HEK-293T (Syn1-Flag)
Cytosol
Membrane
WT
ZDHHC5-OE
ZDHHC5-KO
WT
ZDHHC5-OE
ZDHHC5-KO
Flag
Na+/K+ ATPase
HSP-90
Total
Flag
B
β-actin-flag
Syn1-his
YFP
1 YFPn-Syn1-WT-his
2 YFPn-Syn1-3CA-his
3 YFPC-β-actin-flag
4 Syn1-WT-YFPn
5 Syn1-3CA-YFPn
6 β-actin-flag-YFPC
C
β-actin-flag
Syn1-his
YFP
Merged
1+3
4+3
1+6
4+6

FIG. S6

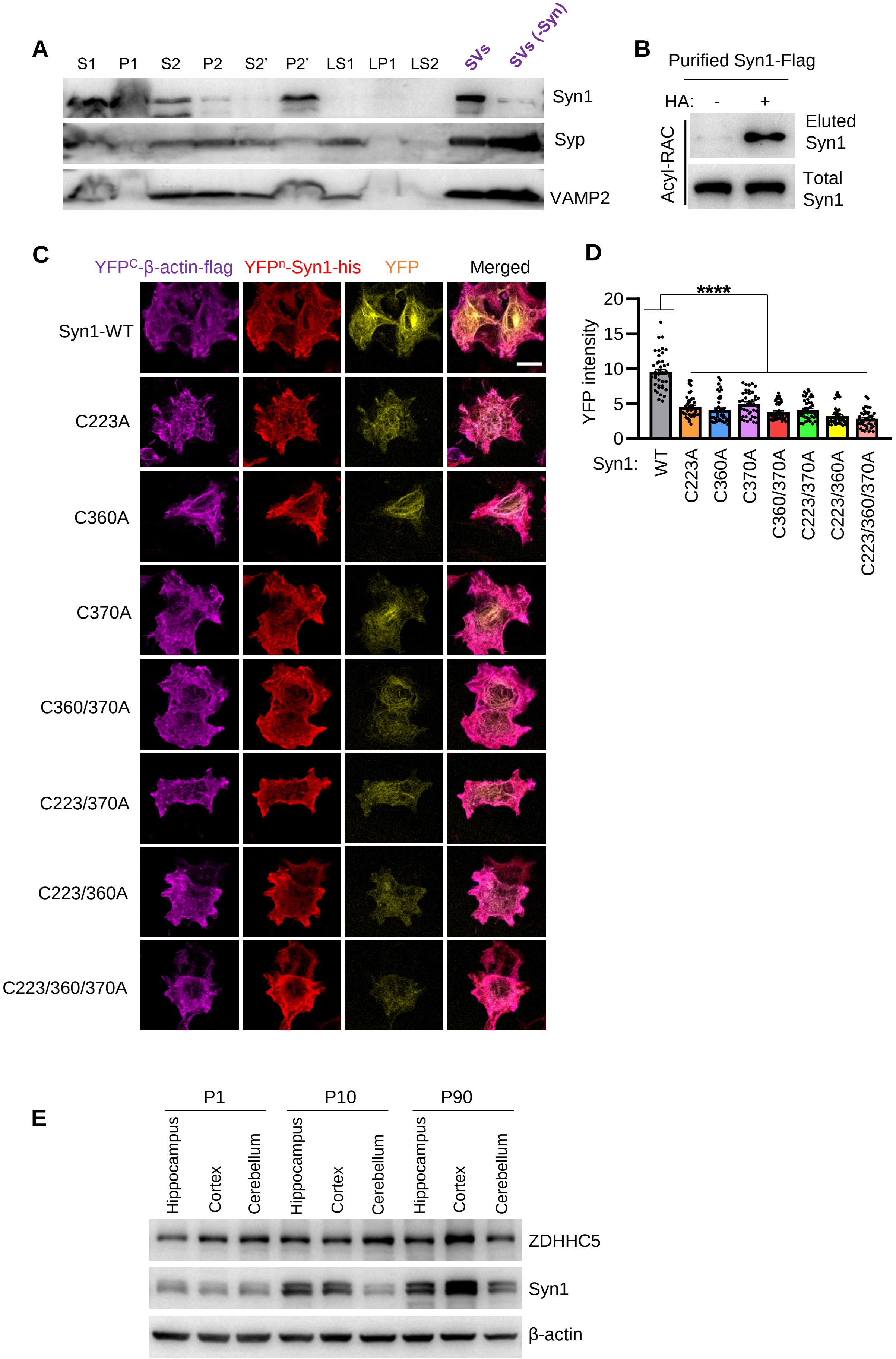
A
S1
P1
S2
P2
S2'
P2'
LS1
LP1
LS2
SVs
SVs (-Syn)
Syn1
Syp
VAMP2
B
Purified Syn1-Flag
HA: - +
Acyl-RAC
Eluted Syn1
Total Syn1
C
YFPC-β-actin-flag
YFPn-Syn1-his
YFP
Merged
Syn1-WT
C223A
C360A
C370A
C360/370A
C223/370A
C223/360A
C223/360/370A
D
****
YFP intensity
20
15
10
5
0
Syn1:
WT
C223A
C360A
C370A
C360/370A
C223/370A
C223/360A
C223/360/370A
E
P1
P10
P90
Hippocampus
Cortex
Cerebellum
Hippocampus
Cortex
Cerebellum
Hippocampus
Cortex
Cerebellum
ZDHHC5
Syn1
β-actin

FIG. S7

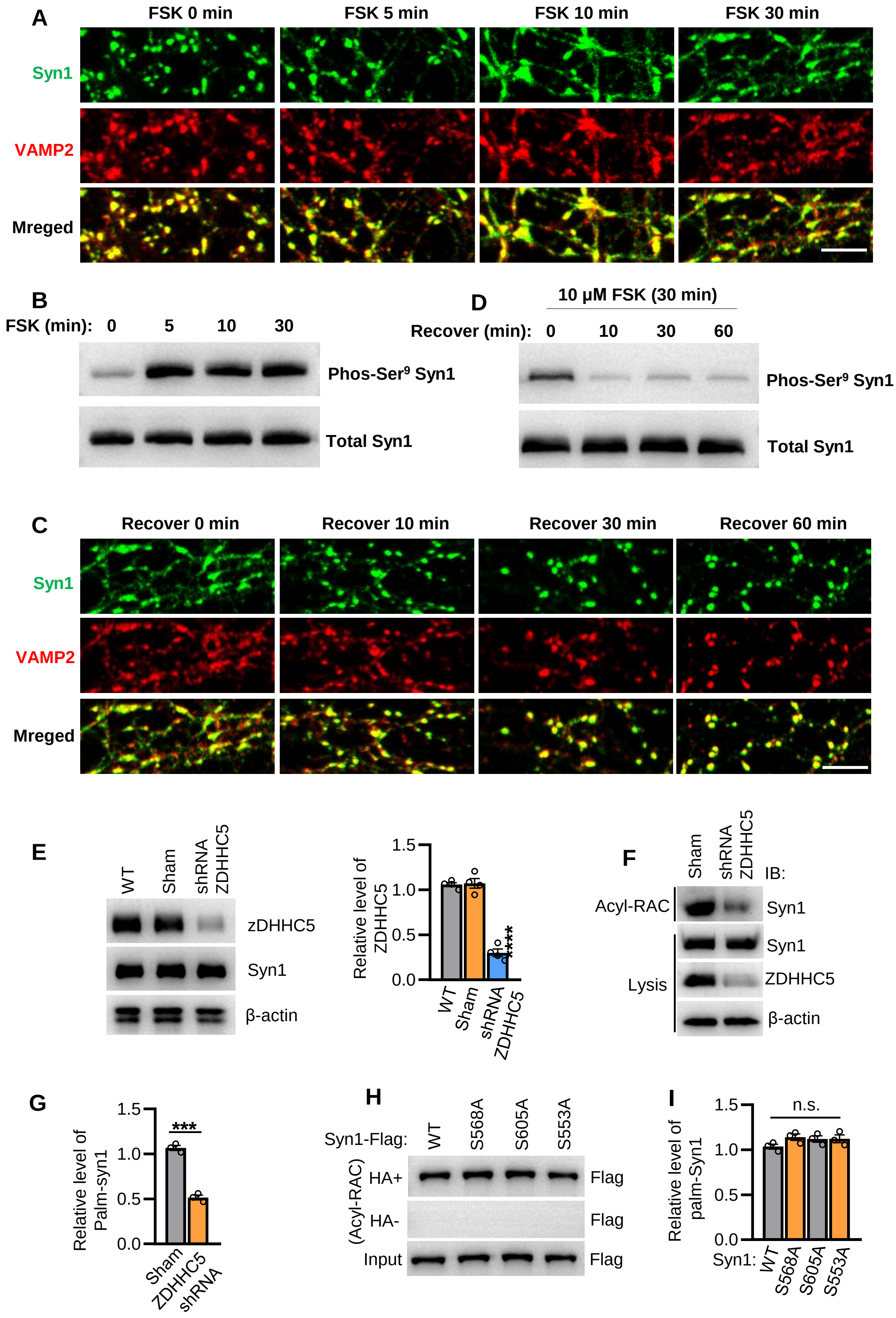
A
FSK 0 min
FSK 5 min
FSK 10 min
FSK 30 min
Syn1
VAMP2
Mreged
B
FSK (min): 0 5 10 30
Phos-Ser9 Syn1
Total Syn1
D
10 μM FSK (30 min)
Recover (min): 0 10 30 60
Phos-Ser9 Syn1
Total Syn1
C
Recover 0 min
Recover 10 min
Recover 30 min
Recover 60 min
Syn1
VAMP2
Mreged
E
WT
Sham
shRNA ZDHHC5
zDHHC5
Syn1
β-actin
Relative level of ZDHHC5
1.5
1.0
0.5
0.0
****
WT
Sham
shRNA ZDHHC5
F
Sham
shRNA ZDHHC5
IB:
Acyl-RAC
Syn1
Syn1
Lysis
ZDHHC5
β-actin
G
Relative level of Palm-syn1
1.5
1.0
0.5
0.0
***
Sham
ZDHHC5 shRNA
H
Syn1-Flag:
WT
S568A
S605A
S553A
(Acyl-RAC)
HA+
HA-
Input
Flag
Flag
Flag
I
Relative level of palm-Syn1
1.5
1.0
0.5
0.0
n.s.
Syn1:
WT
S568A
S605A
S553A

FIG. S8

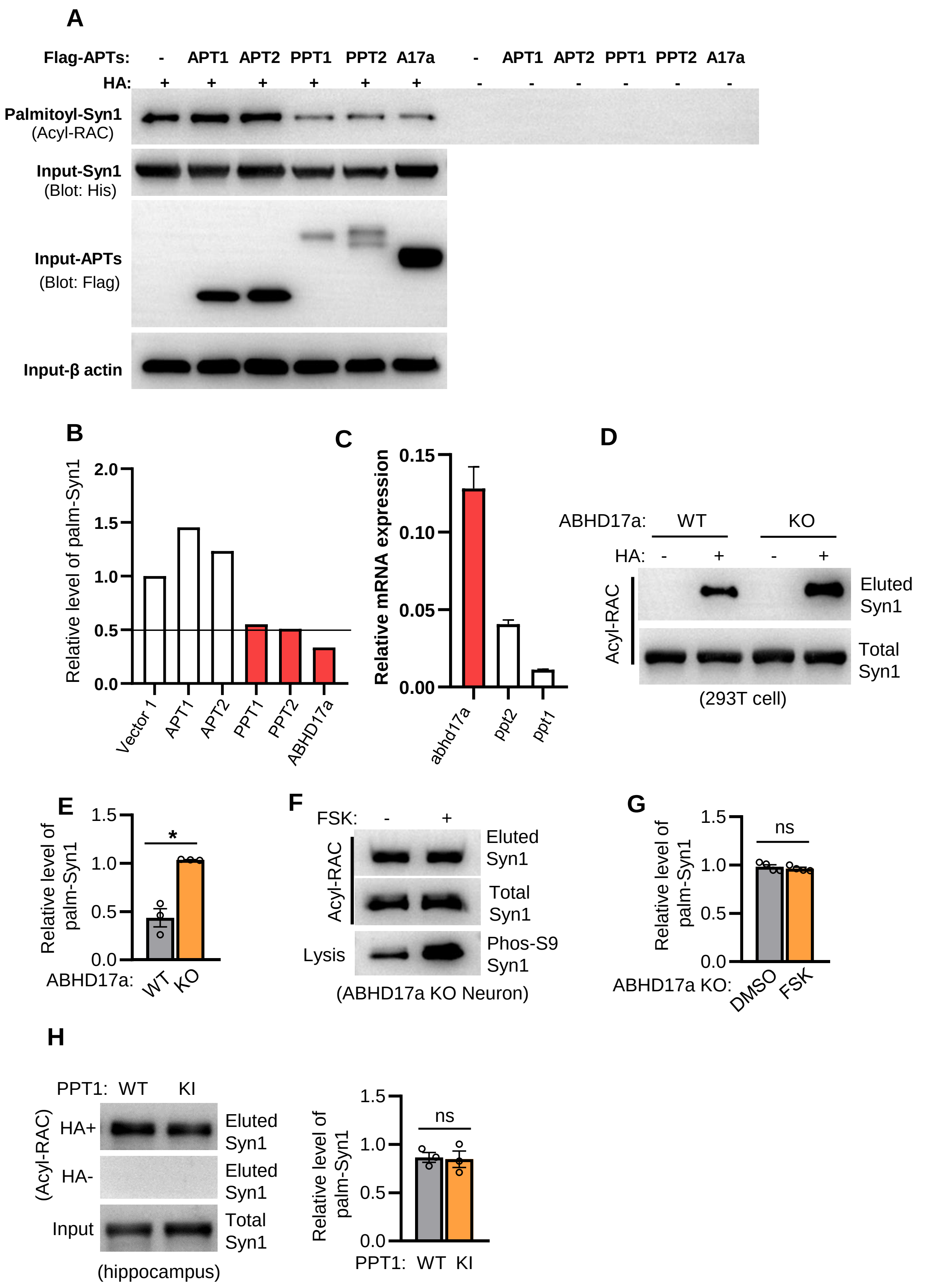
A
Flag-APTs: - APT1 APT2 PPT1 PPT2 A17a - APT1 APT2 PPT1 PPT2 A17a
HA: + + + + + + - - - - - -
Palmitoyl-Syn1
(Acyl-RAC)
Input-Syn1
(Blot: His)
Input-APTs
(Blot: Flag)
Input-β actin
B
Relative level of palm-Syn1
2.0
1.5
1.0
0.5
0.0
Vector 1
APT1
APT2
PPT1
PPT2
ABHD17a
C
Relative mRNA expression
0.15
0.10
0.05
0.00
abhd17a
ppt2
ppt1
D
ABHD17a: WT KO
HA: - + - +
Acyl-RAC
Eluted Syn1
Total Syn1
(293T cell)
E
Relative level of palm-Syn1
1.5
1.0
0.5
0.0
*
ABHD17a: WT KO
F
FSK: - +
Acyl-RAC
Eluted Syn1
Total Syn1
Lysis
Phos-S9 Syn1
(ABHD17a KO Neuron)
G
Relative level of palm-Syn1
1.5
1.0
0.5
0.0
ns
ABHD17a KO: DMSO FSK
H
PPT1: WT KI
(Acyl-RAC)
HA+
HA-
Input
Eluted Syn1
Eluted Syn1
Total Syn1
(hippocampus)
Relative level of palm-Syn1
1.5
1.0
0.5
0.0
ns
PPT1: WT KI

FIG. S9

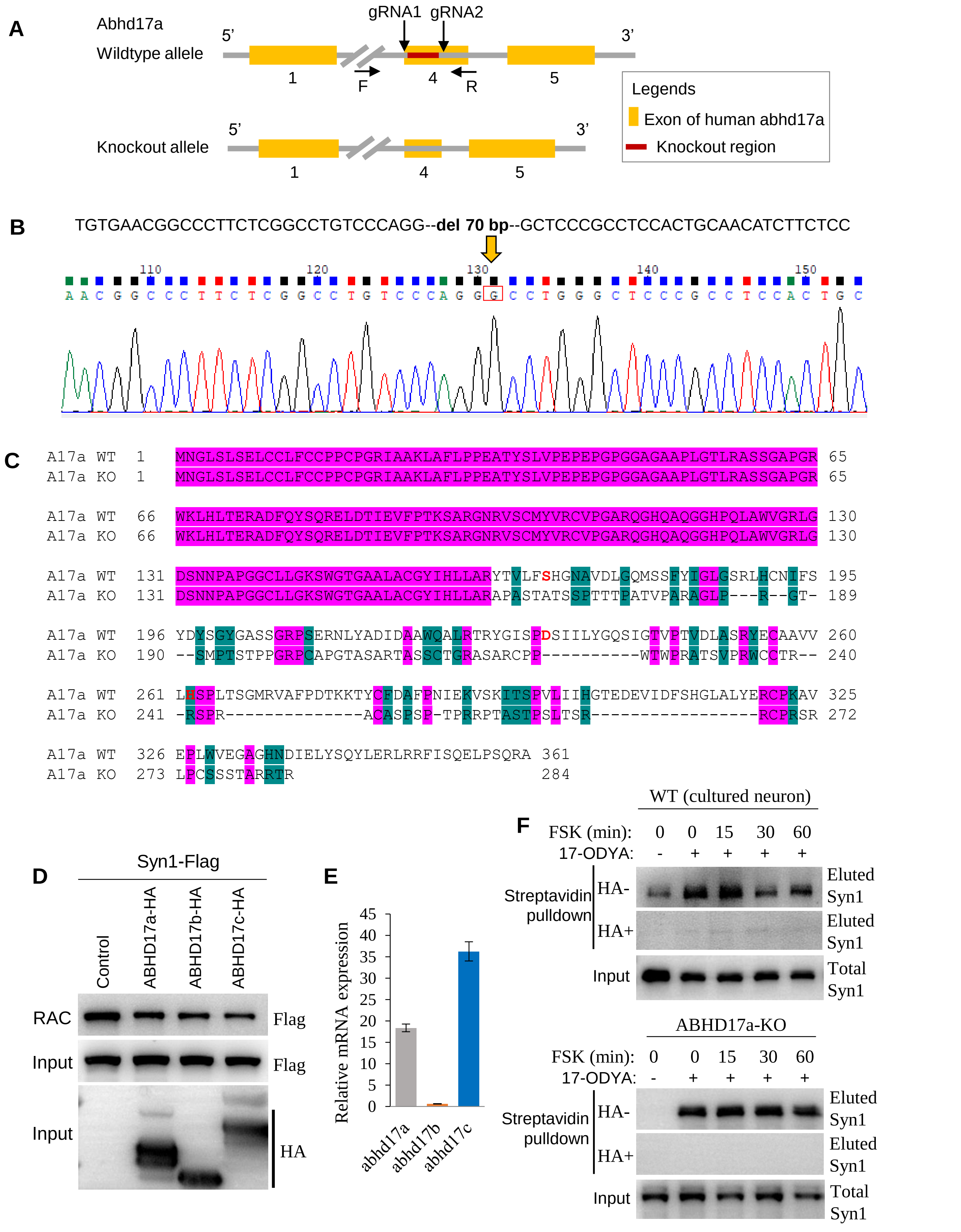
A
Abhd17a
Wildtype allele
gRNA1 gRNA2
5'
3'
1
F
4
R
5
Knockout allele
5'
3'
1
4
5
Legends
Exon of human abhd17a
Knockout region
B
TGTGAACGGCCCTTCTCGGCCTGTCCCAGG--del 70 bp--GCTCCCGCCTCCACTGCAACATCTTCTCC
110
120
130
140
150
C
A17a WT 1 MNGLSLSELCCLFCCPPCPGRIAAKLAFLPPEATYSLVPEPEPGPGGAGAAPLGTLRASSGAPGR 65
A17a KO 1 MNGLSLSELCCLFCCPPCPGRIAAKLAFLPPEATYSLVPEPEPGPGGAGAAPLGTLRASSGAPGR 65
A17a WT 66 WKLHLTERADFQYSQRELDTIEVFPTKSARGNRVSCMYVRCVPGARQGHQAQGGHPQLAWVGRLG 130
A17a KO 66 WKLHLTERADFQYSQRELDTIEVFPTKSARGNRVSCMYVRCVPGARQGHQAQGGHPQLAWVGRLG 130
A17a WT 131 DSNNPAPGGCLLGKSWGTGAALACGYIHLLARYTVLFSHGNAVDLGQMSSFYIGLGSRLHCNIFS 195
A17a KO 131 DSNNPAPGGCLLGKSWGTGAALACGYIHLLARAPASTATSSPTTTPATVPARAGLP---R--GT- 189
A17a WT 196 YDYSGYGASSGRPSERNLYADIDAAWQALRTRYGISPDSIILYGQSIGTVPTVDLASRYECAAVV 260
A17a KO 190 --SMPTSTPPGRPCAPGTASARTASSCTGRASARCPP----------WTWPRATSVPRWCCTR-- 240
A17a WT 261 LHSPLTSGMRVAFPDTKKTYCFDAFPNIEKVSKITSPVLIIHGTEDEVIDFSHGLALYERCPKAV 325
A17a KO 241 -RSPR--------------ACASPSP-TPRRPTASTPSLTSR-----------------RCPRSR 272
A17a WT 326 EPLWVEGAGHNDIELYSQYLERLRRFISQELPSQRA 361
A17a KO 273 LPCSSSTARRTR 284
D
Syn1-Flag
Control
ABHD17a-HA
ABHD17b-HA
ABHD17c-HA
RAC
Flag
Input
Flag
Input
HA
E
Relative mRNA expression
45
40
35
30
25
20
15
10
5
0
abhd17a
abhd17b
abhd17c
F
WT (cultured neuron)
FSK (min): 0 0 15 30 60
17-ODYA: - + + + +
Streptavidin pulldown
HA-
HA+
Eluted Syn1
Eluted Syn1
Input
Total Syn1
ABHD17a-KO
FSK (min): 0 0 15 30 60
17-ODYA: - + + + +
Streptavidin pulldown
HA-
HA+
Eluted Syn1
Eluted Syn1
Input
Total Syn1

FIG. S10

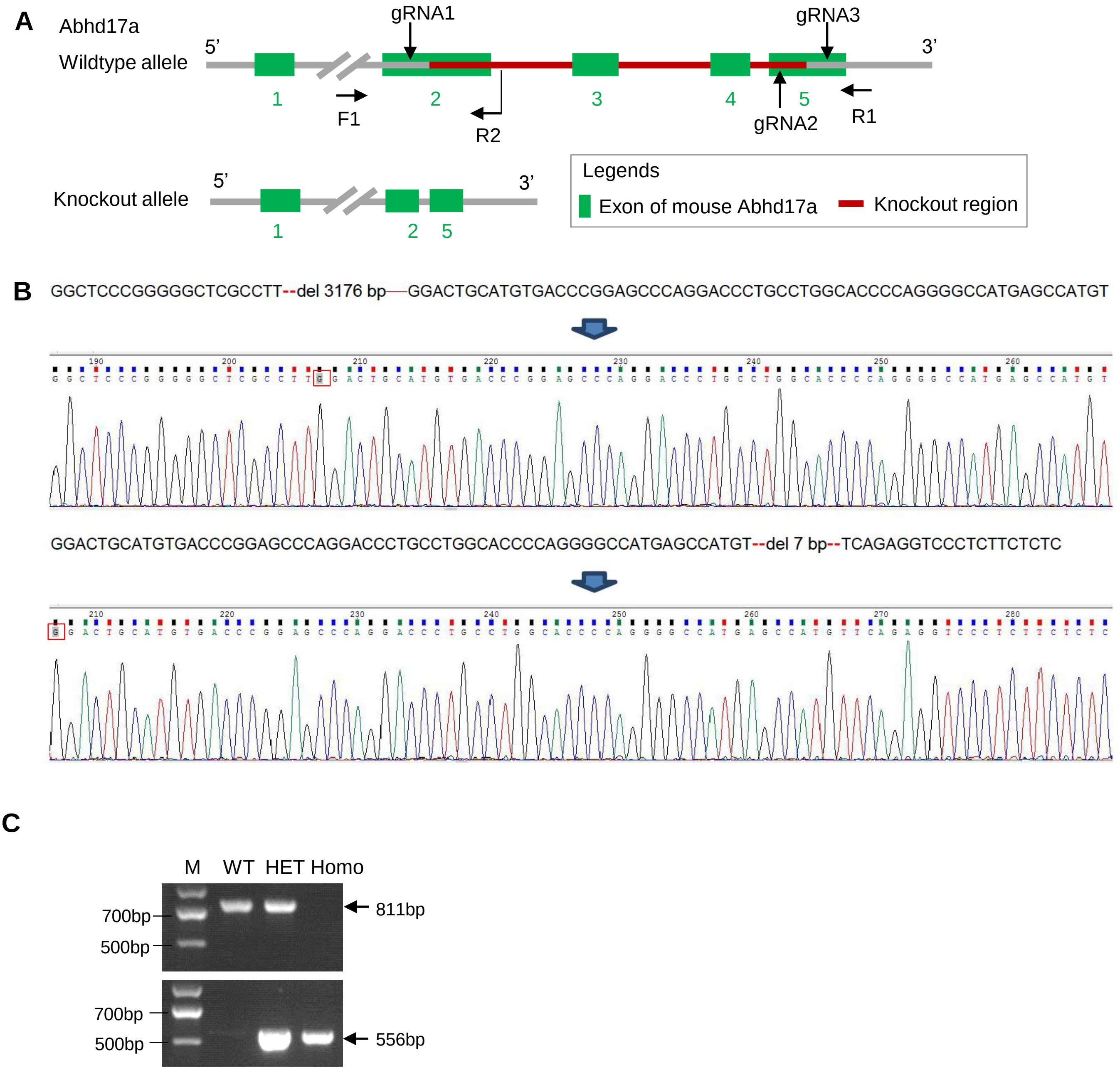
A
Abhd17a
Wildtype allele
gRNA1
gRNA3
5’
3’
1
2
3
4
5
F1
R2
gRNA2
R1
Knockout allele
5’
3’
1
2
5
Legends
Exon of mouse Abhd17a
Knockout region
B
GGCTCCCGGGGGCTCGCCTT--del 3176 bp—GGACTGCATGTGACCCGGAGCCCAGGACCCTGCCTGGCACCCCAGGGGCCATGAGCCATGT
GGACTGCATGTGACCCGGAGCCCAGGACCCTGCCTGGCACCCCAGGGGCCATGAGCCATGT--del 7 bp--TCAGAGGTCCCTCTTCTCTC
C
M
WT
HET
Homo
700bp
500bp
811bp
556bp

Supplemental table 1. The primer sequences used in relative quantitative PCR.

Refseq gene	Accession number	Forward primer (5′-3′)	Reverse primer (5′-3′)	Amplicon size
gapdh	NM_008084	TGACCTCAACTACATGGTCTACA	CTTCCCATTCTCGGCCTTG	85
actb	NM_007393	GGCTGTATTCCCCTCCATCG	CCAGTTGGTAACAATGCCATGT	154
zdhhc1	NM_175160	ATGAACATCTGCAACAAACCCT	GCTCCATCCATTCCTTCGAGAG	126
zdhhc2	NM_178395	GTCAAGGTGATGGCTGTTCC	GATGGCTCTGGGACTCTCTC	147
zdhhc3	NM_178395	ATCCCCACCCATCACTTCC	CTCGGATAAACCACATGGCTC	112
zdhhc4	NM_026917	TTACCTAGATGACGTGGGGC	AAACGATGACAAAGCCCAGT	110
zdhhc5	NM_028379	CCGCCATATTTCTAGTGGGA	TTGCATTGTAAATGGGCACT	99
zdhhc6	NM_144887	GAGTAAGAGGGTGGTTTCCTAGA	GCTGGATCTGAGTCACCATCAC	67
zdhhc7	NM_025883	CACCAGGAGCCTCAGCACT	AGCATCATGGGAGCACTTGT	110
zdhhc8	NM_133967	GGTTGGTTCCAGCACACTCT	AGAGGAAGAGGATGCCATTG	98
zdhhc9	NM_172151	GGGCATCTTCTACCTGACCC	AGACAGCTGAACAGCCAGGT	91
zdhhc11	NM_027704	GCCGAAGTCAAGTGTGTGTT	GCACCACAGAGTAGAAGGGT	145
zdhhc12	NM_025428	GGGAATCACTCTGGTGCTCT	CCCCTTGCTCTTCCCATT	104
zdhhc13	NM_028031	TGGTTCTAGCCTGGACATCC	GCCAAAGCCGAAACTGTTTG	137
zdhhc14	NM_146073	CGGCGTCTTCTACCTGACTC	GATGGCAGGGGTGATCTTCT	100
zdhhc15	NM_175358	TGCCAGTGCTCGTTATTGTC	AACTTTTTCCGCTGGACTCA	98
zdhhc16	NM_023740	TACAGCTGCCAGCCTTTCC	CCCAACAGCAGACTTCGC	115
zdhhc17	NM_172554	GCGGGAGGAGGGATTTAACAC	CCCGTTTCGGTCTCGTACTC	63
zdhhc18	NM_001017968	TCAACGGGCAGACAGTGAAAC	GAAGCGGTAGTTCCGTCTCC	158
zdhhc19	NM_199309	TGTGACACTTGTGAAGGAACC	AAAAACAGCAGCAGCGTTACA	106
zdhhc20	NM_029492	ACCTTTGTGGTCGTCTGGTC	GCCACAAGGTAAACAACGGT	104
zdhhc21	NM_026647	GCTGCTTACTTGCTACGCAC	CTCATGGCGAACAACAAAGA	100
zdhhc22	NM_001080943	CGGCTGCTCAACGTGGTAG	CCAGGACGTAATTGCCCAGG	190
zdhhc23	NM_001007460	GGCTGCCTGTTTGTGTGATTG	CCGTGATTCTTTCGCAAGTCTC	98
zdhhc24	NM_027476	ACAGTGGCTCTCCTGCTGTT	CACACGTGTCCACCACAAA	94
zdhhc25	NM_027306	TTGGACTTACCTCGACCCAC	ATAGGGGCAGGTAGGGACAC	109
ppt1	NM_008917.3	GTGGCCAAGCTAAGGAAACC	GTCCCCTTCCTTAGCCAGAA	119
ppt2	NM_019441.5	AAGAGACCATCCCAATGCCA	AGCTAGATTGCCAGGGAGTG	110
lypla1	NM_008866	GCCTTTAATCCCAGCACTCG	GGGTTTCTCTGTGTAGCCCT	103
lypla2	NM_011942	ACTTCTGCCTCCGGTCTAAC	TAGGGTGGGAAGAGAAGGGA	132
abhd17a	NM_145421.2	TCCCTGACACCAAGAAGACC	CCCGTGAGAGAAGTCGATCA	125
abhd17b	NM_146096.3	TTCTGCTGTCCACCTTGC	AACGCACAAACATACAAGCA	220
abhd17c	NM_133722.2	CTTACGACTACTCGGGCTATG	CACGCAAACCAGACATCAG	228
