# Supplementary material for: Crosstalk of Synapsin1 palmitoylation and phosphorylation controls the dynamicity of synaptic vesicles in neurons
Source: Cell Death Dis. 2022 Sep 12;13(9):786. doi: 10.1038/s41419-022-05235-4 (PMC9468182; doi:10.1038/s41419-022-05235-4)
Supplement: Supplementary file 2 — Supplemental figure legends [file 41419_2022_5235_MOESM2_ESM.pdf]

Supplemental figure legends

Fig. S1 Syn1 is palmitoylated at Cys-223, Cys-360 and Cys-370. A, HEK-293T cells expressing Syn1-Flag were metabolically labeled with 17-ODYA and the lysates were reacted with biotin-azide and enriched with streptavidin-agarose beads, hydroxylamine (HA) was used to hydrolyze thioester linkage and remove 17-ODYA labeling. **B**, Syn1-flag was purified by anti-DYKDDDDK affinity resin and processed with SDS-PAGE and Coomassie blue staining. **C**-**D**, purified Syn1-flag was probed by mass-spectrometry, a mass-shift of 238 Da linked to cysteine is a hallmark for palmitoylation.

Fig. S2 Generation of Syn1-KO mice. A, Targeting scheme of the truncation of exons 2-5 in mouse Syn1. Accordingly, two guide RNAs of syn1 (GTGGACAGTTGCGTCTGAATAGG, GATTTTGAAATATTCGTATAAGG) were designed. **B**, the knockout allele has a deletion of 3440 bp, confirmed by DNA sequencing. **C**, genotyping was carried out by PCR amplification with corresponding primers and the amplicons of WT and knockout alleles are 498bp and 630 bp, respectively. **D**, WB confirmed that Syn1 is deleted in Syn1-KO mice brain.

Fig. S3 ZDHHC5 possibly palmitoylates Syn1. A-B, HEK-293T cells were transfected with Syn1-his and each ZDHHCs, then the level of palm-Syn1 is evaluated and quantified (**B**). **C**, the relative mRNA levels of ZDHHCs expressed in adult mice hippocampus were analyzed by Real-Time PCR. GAPDH was used as an internal control.

Fig. S4 Deleting ZDHHC5 in HEK-293T cells or in mice. A, Targeting scheme of the truncation of exons 4 in human ZDHHC5. Two sgRNAs were designed to target the enzyme activity center (GCATCCAGGTGCGCATGAAATGG, ATGAAGTCTTACCCCACCCAGGG) of human zdhhc5. **B**, the knockout allele has a deletion of 75 bp on exon 4 and 13 bp on intron 4, confirmed by sequencing. **C**, ORF finder predicts that the knockout allele results in a deletion of 25 aa in its activity center.

Cysteine residues labeled in red are essential for its enzyme activity. **D**, Targeting scheme of the truncation of exons 5-11 in mouse ZDHHC5. Accordingly, two sgRNAs (TTGTAAGCTGATTGATGTACAGG, GCTTAATTAGTGAAGGCATCAGG) were designed. **E**, the knockout allele has a deletion of 5885 bp, confirmed by sequencing. **F**, genotyping was carried out by PCR amplification with corresponding primers and the amplicons of WT and knockout alleles are 729 bp and 488 bp, respectively. **G**, WB confirmed that ZDHHC5 is deleted in ZDHHC5-KO mice brain.

Fig. S5 Syn1 interacts with β-actin ex vivo. A, ZDHHC5 was either overexpressed or depleted in HEK-293T cells expressing Syn1-Flag, which were then processed for cytosol and membrane fractionations. **B**, YFP^n was tagged at either the N-terminus or C-terminus of Syn1 (WT and Syn1-3CA) and YFP^c was tagged to β-actin, both of which were transfected into HEK-293T cell for BiFC assay. When expressed alone, none of them excites YFP fluorescence. **C**, Only the coexpression of YFP^n-Syn1 and YFP^c-β-actin (1+3) could activate YFP fluorescence, but not other setups.

Fig. S6 Palmitoylation is involved in regulating Syn1-F-actin binding. A, Enrichment of SVs from WT mice hippocampi, 'SVs' is the final enrichment of synaptic vesicles characterized by SV marker proteins (Syn1, Syp and VAMP2) and after NaCl treatment (0.5 M) Syn is depleted from SVs to obtain the 'SVs (-Syn)'. **B**, Syn1-Flag was expressed in HEK-293T cells and purified by Flag antibody, which was then examined by Acyl-RAC assay. **C-D**, YFP^n was tagged to Syn1-WT or its mutants and YFP^c was tagged to β-actin, both of which were transfected into HEK-293T cell for BiFC assay (**C**) and the intensity of YFP fluorescence was quantified accordingly (n=43 cells from 4 repeats, one-way ANOVA, ****$p < 0.0001$) (**D**). **E**, WT mice brains were collected at different developmental stages and analyzed for the expressions of ZDHHC5 and Syn1. Data are mean ± s.e.m.

Fig. S7 FSK triggers Syn1 phosphorylation and SVs release. A, Hippocampal neurons (DIV15) were treated with FSK (10 μM) for various periods and fixed for

imaging. VAMP2 is a marker for SV cluster. Scale bar, 5 μm. **B**, FSK treated neurons were examined for the level of phosphor-Syn1 (S9). **C**, After FSK treatment, FSK was washed out and allowed to recover for different periods, and then fixed for imaging. Scale bar, 5 μm. **D**, the level of phosphor-Syn1 (S9) was evaluated after FSK washout. **E**, Hippocampal neurons were infected with Sham or ZDHHC5 shRNA lentivirus and evaluated for the level of ZDHHC5. **F-G**, Hippocampal neurons were infected with Sham or ZDHHC5 shRNA and evaluated for the level of palm-Syn1 (n=3, ***p=0.001). **H-I**, Syn1 and its mutants (S568A, S605A and S553A) were expressed in HEK-293T cells and evaluated for the level of palm-Syn1 (n=3, p=0.2466). n.s., not significant. Data are mean ± s.e.m.

Fig. S8 ABHD17a potentially depalmitoylates Syn1. A-B, Syn1-his was expressed with individual thioesterases in HEK-293T cells for the evaluation of the level of palm-Syn1. **C**, the relative mRNA levels of abhd17a, ppt1 and ppt2 in the hippocampus of adult mice, analyzed by Real-Time PCR. **D-E**, Syn1-his was expressed in WT and ABHD17a-KO HEK-293T cells and evaluated for the level of palm-Syn1 (n=3, *p=0.0237). **F-G**, hippocampal neurons isolated from ABHD17a-KO mice were treated with or without FSK (10 μM) for 30 min and probed for the level of phospho-Syn1 and palm-Syn1 (n=4, p=0.4754). **H-I**, Hippocampal lysates of WT and PPT1-KI mice were examined for the level of palm-Syn1 (n=3, p=0.8632). n.s., not significant. Data are mean ± s.e.m.

Fig. S9 Deleting ABHD17a in HEK-293T cell. A, targeting scheme of the truncation of exons 4 in human ABHD17a. Accordingly, two sgRNAs were designed (GAGAAGAGGACCGTGTACCTGGG, GAGCAGCTTCTACATTGGCCTGG). **B**, the knockout allele has a deletion of 70 bp, confirmed by DNA sequencing. **C**, The ORF finder predicts that the knockout allele causes a frameshift transcription. The S170/D235/H264 are the catalytic triad (labeled in red), which are essential for its enzyme activity. **D**, ABHD17a, ABHD17b or ABHD17c was coexpressed with Syn1-Flag in HEK-293T cells for the evaluation of palm-Syn1. **E**, the relative mRNA

expression of abhd17a, abhd17b and abhd17c were measured by Real-time PCR. **F**, Cortical neurons prepared from WT and ABHD17a-KO mice were metabolically labeled with 17-ODYA and treated with or without FSK for varied time periods, the lysates of which were reacted with biotin-azide and enriched with streptavidin-agarose beads, hydroxylamine (HA+) was used to hydrolyze thioester linkage and remove 17-ODYA labeling.

Fig. S10 Generation of ABHD17a-KO mice. A, targeting scheme of the truncation of exons 2-5 in mouse ABHD17a. Accordingly, three sgRNAs were designed (GGCTCGCCTTGGACCGCGATGGG, TCACATGCAGTCCCGGCAGGAGG, GAGCCATGTTCAGGCGTCAGAGG) to target exon 2-5 and its activity center. **B**, the knockout allele has a deletion of 3176 bp and 7 bp, confirmed by sequencing. **C**, genotyping was carried out by PCR amplification with corresponding primers and the amplicons of WT and knockout alleles are 811bp and 556 bp, respectively.
