# Supplementary material for: Crosstalk of Synapsin1 palmitoylation and phosphorylation controls the dynamicity of synaptic vesicles in neurons
Source: Cell Death Dis. 2022 Sep 12;13(9):786. doi: 10.1038/s41419-022-05235-4 (PMC9468182; doi:10.1038/s41419-022-05235-4)
Supplement: Supplementary file 3 — Supplementary file 1 [file 41419_2022_5235_MOESM3_ESM.pdf]

Uncropped western blot for Figure 1

Uncropped blot of Fig.1A

HEK 293T

HA: - +

95 kd-
72 kd-

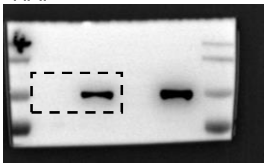

Eluted Syn1 (Blot: his)

HA: - +

95 kd-
72 kd-

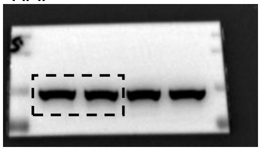

Total Syn1 (Blot: his)

Uncropped blot of Fig.1B

Mice hippocampus

HA: - +

95 kd-
72 kd-

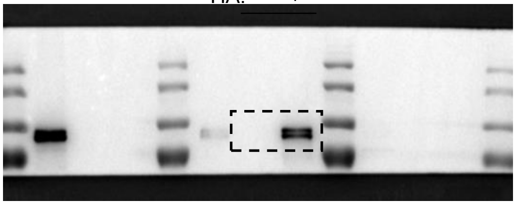

Eluted Syn1 (Blot: Syn1)

HA: - +

95 kd-
72 kd-

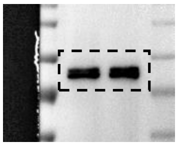

Total Syn1 (Blot: Syn1)

Uncropped blot of Fig.1C

2BP: - - + +
HA: - + - +

95 kd-
72 kd-

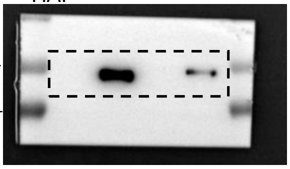

Eluted Syn1 (Blot: his)

2BP: - - + +
HA: - + - +

95 kd-
72 kd-

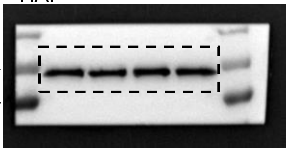

Total Syn1 (Blot: his)

Uncropped blot of Fig.1G

Syn1-WT Syn1-3CA Syn1-C223A Syn1-C360A Syn1-C370A Syn1-WT Syn1-3CA Syn1-C223A Syn1-C360A Syn1-C370A

HA: + + + + + - - - - -

95 kd-
72 kd-

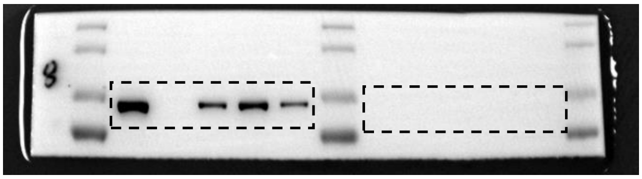

Eluted Syn1 (Blot: his)

Syn1-WT Syn1-3CA Syn1-C223A Syn1-C360A Syn1-C370A

95 kd-
72 kd-

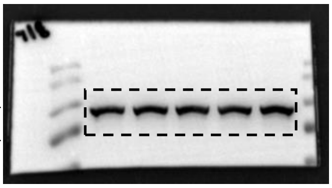

Total Syn1 (Blot: his)

Uncropped western blot for Figure 3

Uncropped blot of Fig. 3A

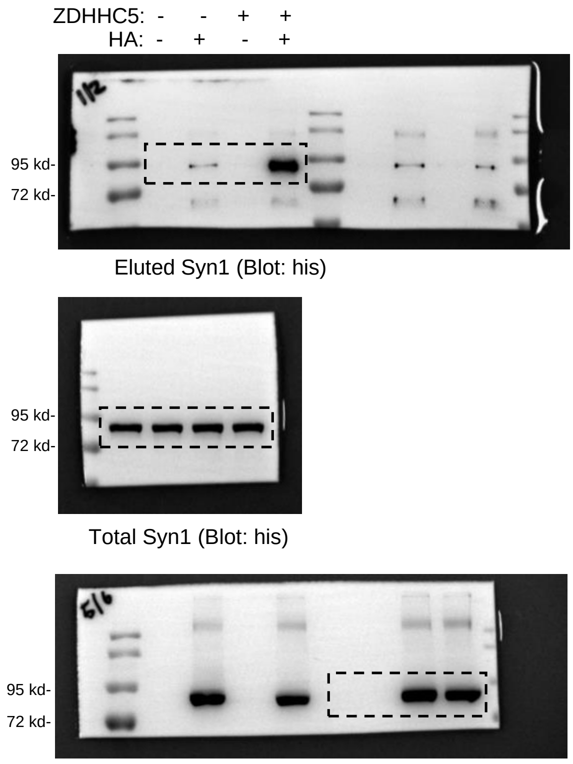

Eluted Syn1 (Blot: his)

Total Syn1 (Blot: his)

Total ZDHHC5 (Blot: flag)

Uncropped blot of Fig. 3E

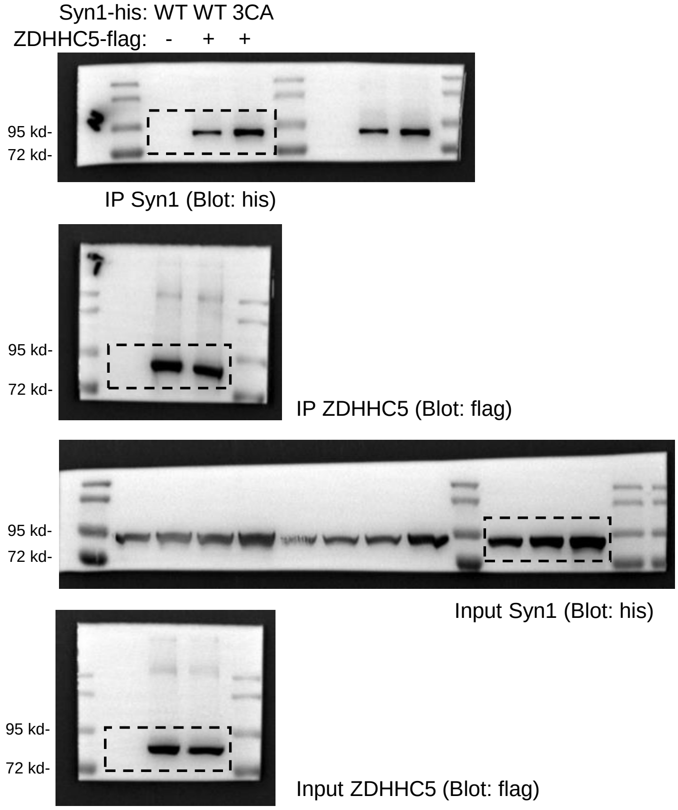

IP Syn1 (Blot: his)

IP ZDHHC5 (Blot: flag)

Input Syn1 (Blot: his)

Input ZDHHC5 (Blot: flag)

Uncropped blot of Fig. 3C

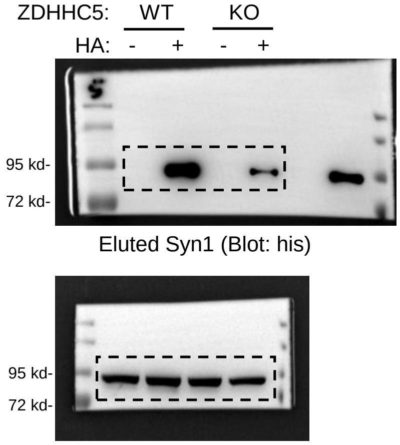

Eluted Syn1 (Blot: his)

Total Syn1 (Blot: his)

Uncropped blot of Fig. 3F

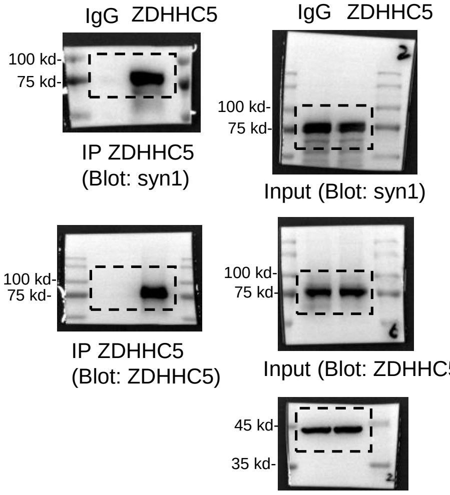

IP ZDHHC5 (Blot: syn1)

Input (Blot: syn1)

IP ZDHHC5 (Blot: ZDHHC5)

Input (Blot: ZDHHC5)

Input (Blot: β-actin)

Uncropped blot of Fig. 3H

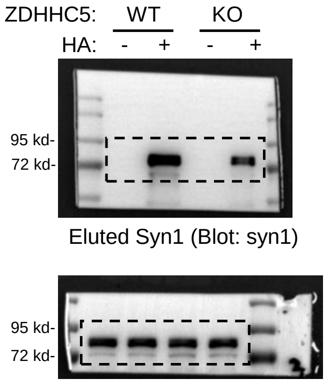

Eluted Syn1 (Blot: syn1)

Total Syn1 (Blot: syn1)

Uncropped western blot for Figure 4

Uncropped blot of Fig.4A

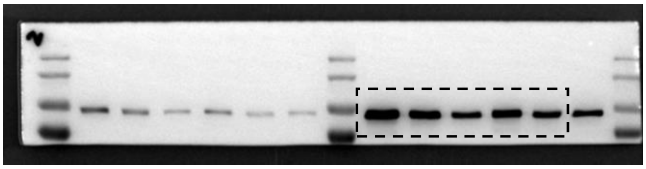

SV-binding syn1 (blot: syn1)

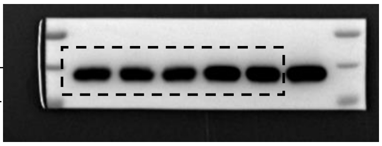

SV-binding syp (blot: syp)

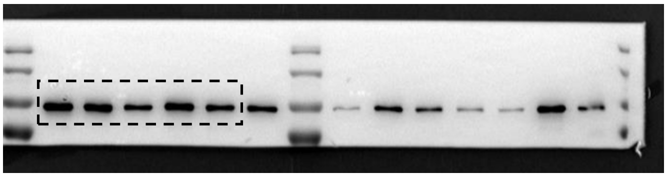

Input syn1 (blot: syn1)

Uncropped blot of Fig. 4C

Syn1-flag: WT 3CA

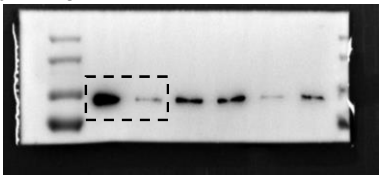

F-actin binding syn1 (blot: flag)

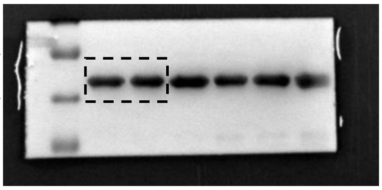

F-actin binding actin (blot: Pan-actin)

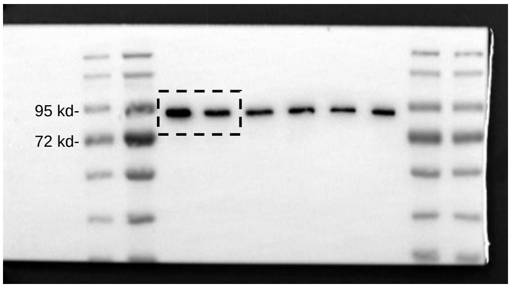

Input syn1 (blot: flag)

Uncropped blot of Fig.4E

Syn1-WT-flag

HA: - +

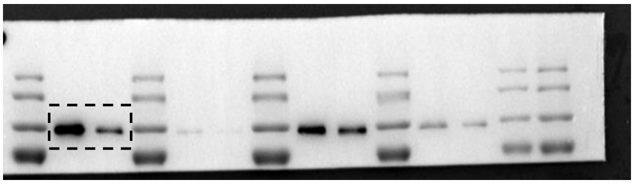

F-actin binding syn1 (blot: flag)

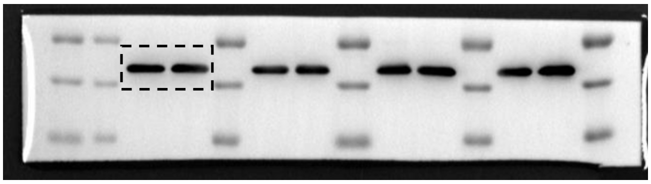

F-actin binding actin (blot: Pan-actin)

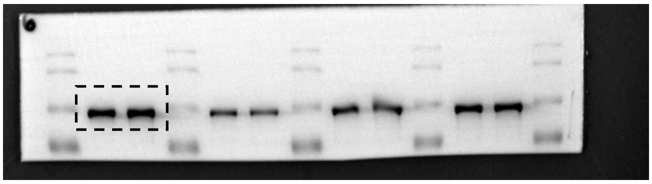

Input syn1 (blot: flag)

Uncropped blot of Fig. 4G

Syn1-C360/370A-flag

HA: - +

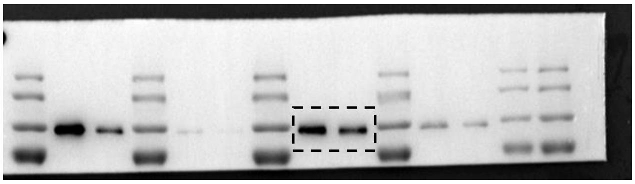

F-actin binding syn1 (blot: flag)

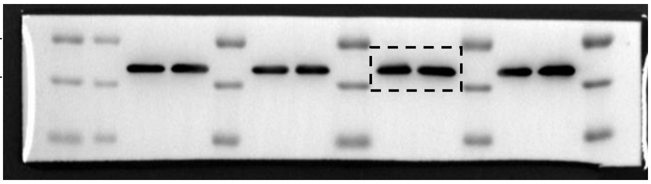

F-actin binding actin (blot: Pan-actin)

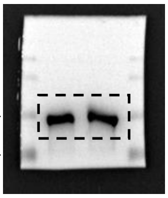

Input syn1 (blot: flag)

Uncropped western blot for Figure 4 continued

Uncropped blot of Fig.4I

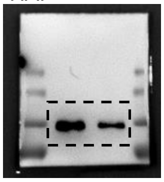

F-actin binding syn1 (blot: flag)

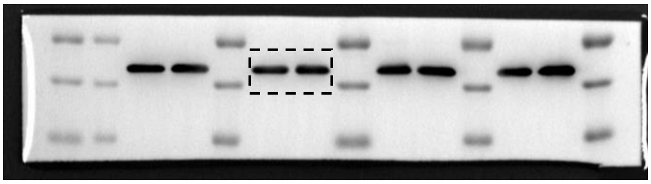

F-actin binding actin (blot: Pan-actin)

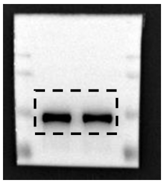

Input syn1 (blot: flag)

Uncropped blot of Fig. 4K

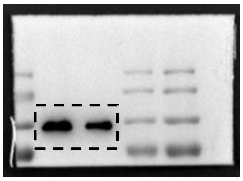

F-actin binding syn1 (blot: flag)

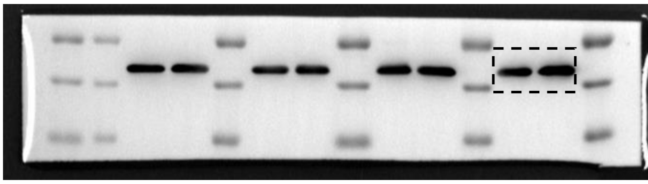

F-actin binding actin (blot: Pan-actin)

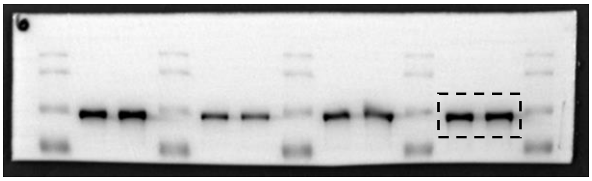

Input syn1 (blot: flag)

Uncropped western blot for Figure 5

Uncropped blot of Fig.5A

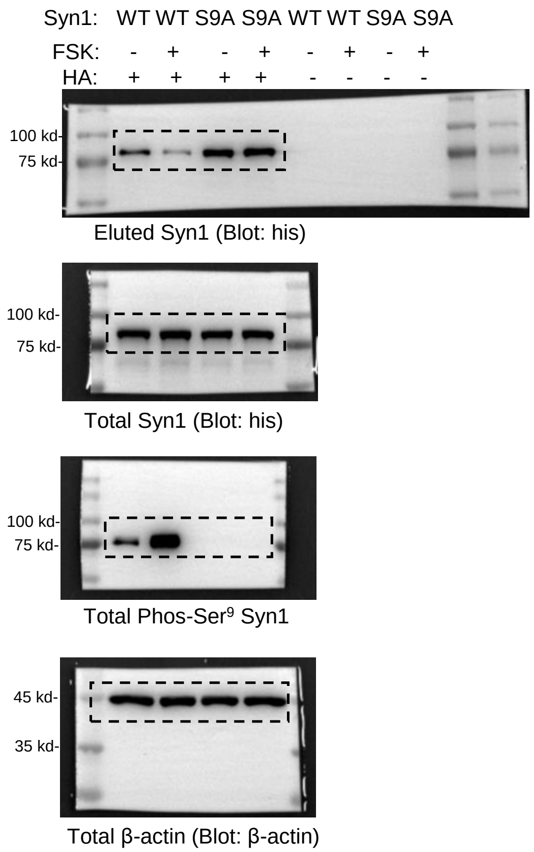

Uncropped blot of Fig.5C

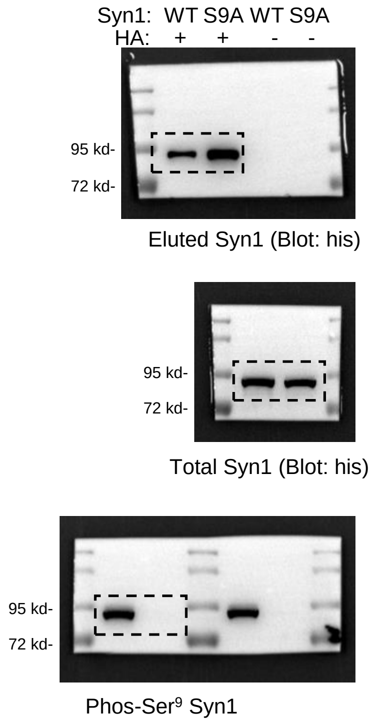

Uncropped blot of Fig.5H

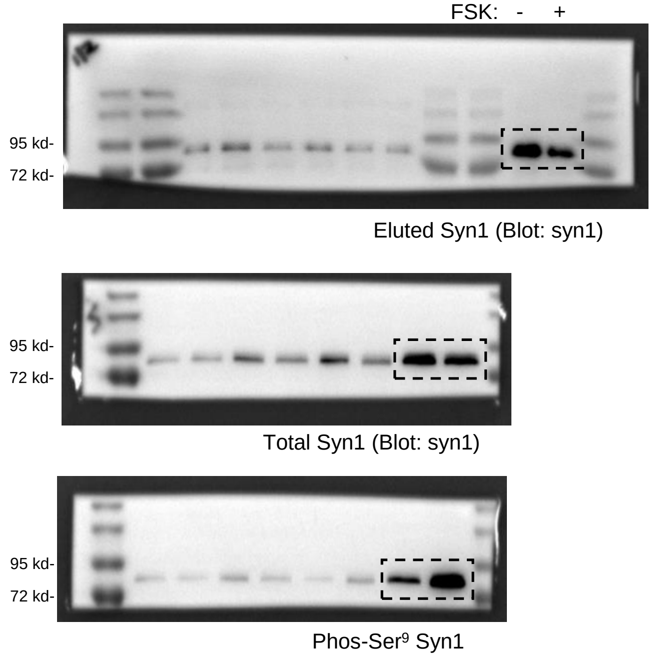

Uncropped blot of Fig.5J

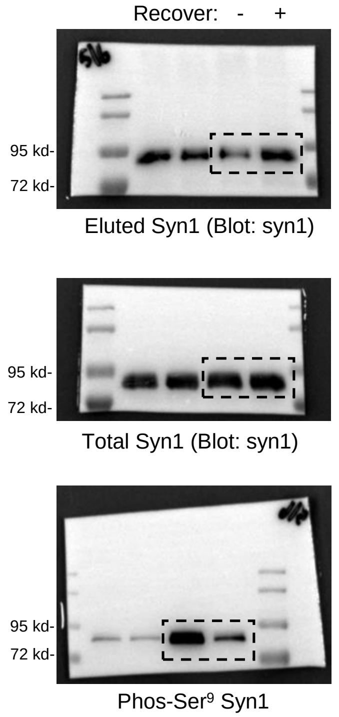

Uncropped western blot for Figure 5 continued

Uncropped blot of Fig.5L

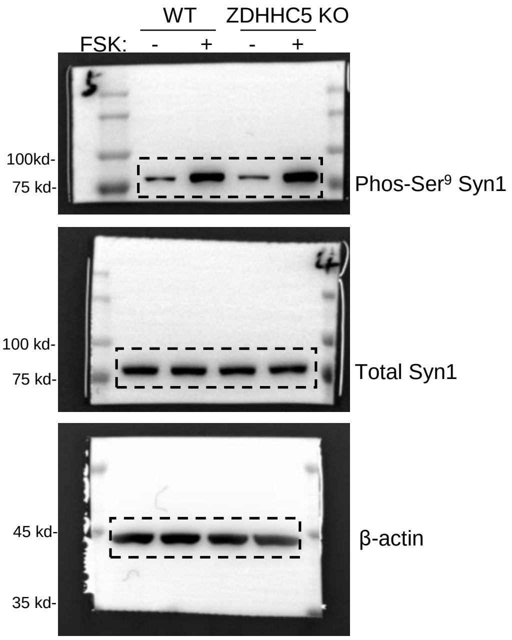

Uncropped blot of Fig.5N

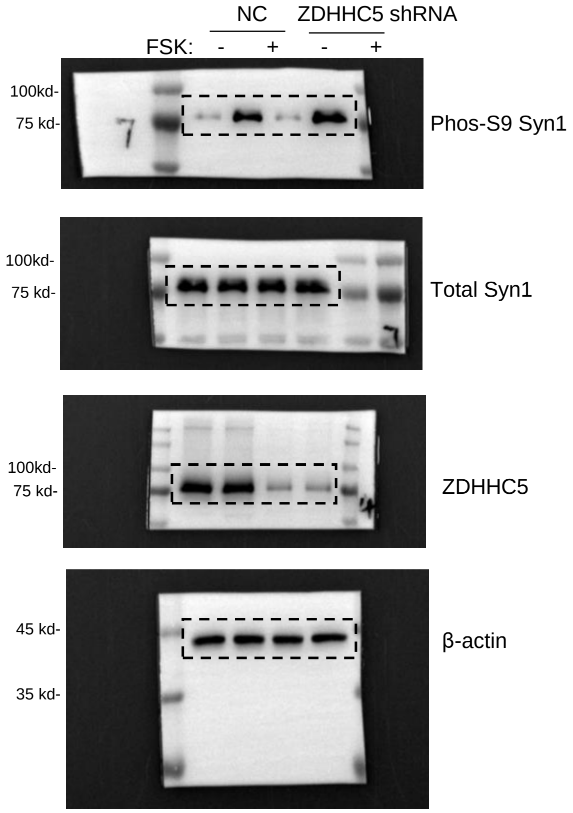

Uncropped blot of Fig.5P

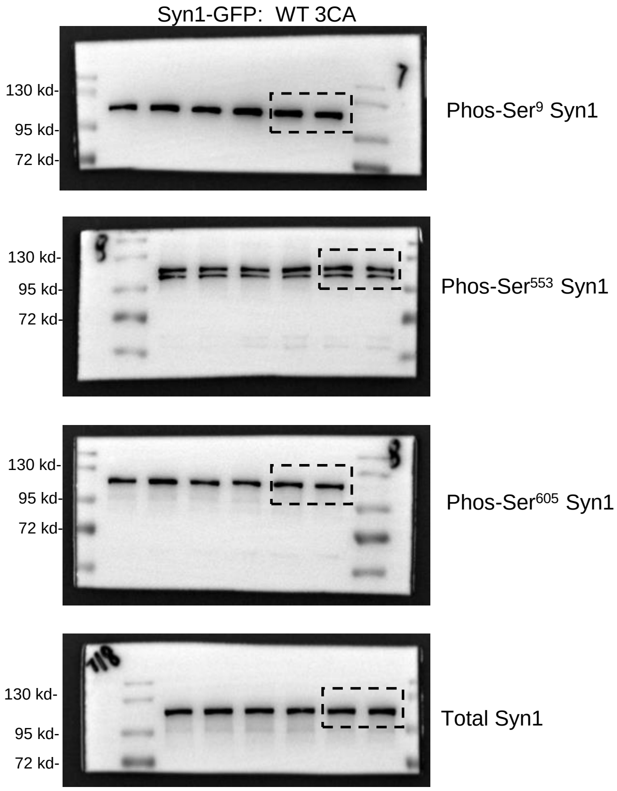

Uncropped western blot for Figure 6

Uncropped blot of Fig.6A

Palm B: - + - + - + - +
FSK: - - + + - - + +
HA: + + + + - - - -

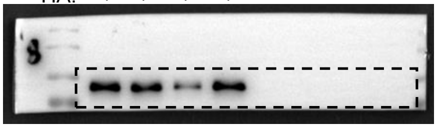

Eluted Syn1 (Blot: syn1)

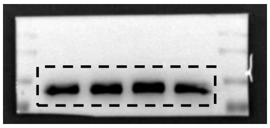

Total Syn1 (Blot: syn1)

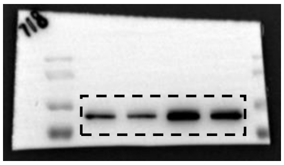

Phos-Ser^{9} Syn1

Uncropped blot of Fig.6E

ABHD17a: - - + +
HA: - + - +

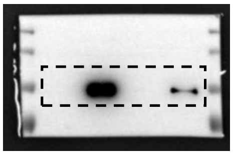

Eluted Syn1 (Blot: his)

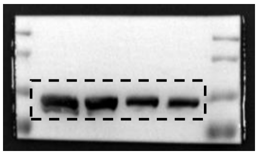

Total Syn1 (Blot: his)

Uncropped blot of Fig.6I

Syn1-his: + +
ABHD17a-flag: - +

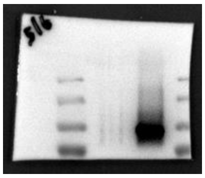

IP Syn1 (Blot: his)

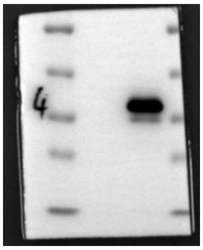

IP ABHD17a (Blot: flag)

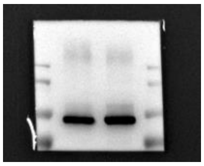

Input Syn1 (Blot: his)

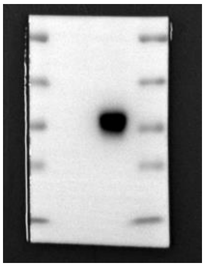

Input ABHD17a (Blot: flag)

Uncropped blot of Fig.6G

ABHD17a: WT KO
HA: - + - +

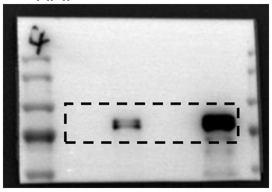

Eluted Syn1 (Blot: syn1)

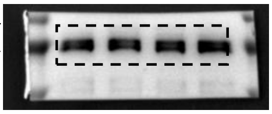

Total Syn1 (Blot: syn1)
